# The complete genome sequence of a bile-isolated *Stenotrophomonas maltophilia* ZT1

**DOI:** 10.1186/s13099-021-00456-y

**Published:** 2021-10-28

**Authors:** Min Zhang, Lixiang Li, Hongwei Pan, Tao Zhou

**Affiliations:** 1 Department of Gastroenterology, Qilu Hospital, Shandong University, 107 Wenhuaxi Road, Shandong 250012 Jinan, People’s Republic of China; 2 Key Laboratory of Translational Gastroenterology, Shandong University, 250012 Jinan, People’s Republic of China; 3 Department of Clinical Laboratory, Qilu Hospital, Shandong University, Shandong 250012 Jinan, China

## Abstract

**Background:**

*Stenotrophomonas maltophilia* is one of the most frequently isolated opportunistic pathogens that can cause infections in humans. Many researches concerned the mechanism of antibiotic resistance displayed by *S. maltophilia*, however, the mechanism of its pathogenesis and its adaptation to special niches, such as bile, remain unclear.

**Results:**

In this study, the *S. maltophilia* strain ZT1 was isolated from human bile. Its genome was sequenced and a circular chromosome of 4,391,471 bp was obtained with a GC content of 66.51%. There were 3962 protein-coding sequences, 7 rRNAs and 74 tRNAs in the chromosome. Compared with Virulence Factor Database, we identified more than 500 candidate virulence genes including genes encoding fimbrial assembly protein, enterobactin synthesis pathway proteins, efflux pumps, and the DNA and/or proteins secretion system in the genome of strain ZT1. Additionally, there were at least 22 genes related to bile adaption, including *emrAB, acrRAB*, *galU*, rfbC, *tolC* and *mdtABC*.

**Conclusions:**

This is the first study to reveal the whole genome sequence of the ZT1 strain of *S. maltophilia* isolated from human bile. We identified hundreds virulence factors and 22 bile adaptation-related genes in the genome of the *S. maltophilia* strain ZT1. Further comparative genomic analysis and functional verification would aid in understanding the pathogenesis and bile adaptation of *S. maltophilia*.

**Supplementary Information:**

The online version contains supplementary material available at 10.1186/s13099-021-00456-y.

## Background

*Stenotrophomonas maltophilia* is the third largest non-fermentative gram-negative bacillus, after *Pseudomonas aeruginosa* and *Acinetobacter baumannii* [1]. It can cause opportunistic infections, especially in hospitalized and immunocompromised patients. In recent year, the number of clinical isolates of *S. maltophilia* has shown an upward trend [2]. Many clinical studies have found that *S. maltophilia* strains are resistant to several antibiotics, making its treatment challenging and infection life-threatening [3]. Currently, *S. maltophilia* is considered an emerging multidrug-resistant, global, and opportunistic pathogen [4].

*S. maltophilia* is a common microbe widely distributed in water, soil, plant-associated habitats, and animal tissues [5]. *S. maltophilia* has been isolated from the sputum, secretion, urine, blood, wound drainage and cerebrospinal fluid of patients [1]. These studies demonstrate the high environmental adaptability of *S. maltophilia* [2]. Currently, there are more than 600 genome assemblies of *S. maltophilia* accessible in the NCBI database. Many genomic studies have focused on the genes related to antibiotic resistance and the virulence of *S. maltophilia* [6–8]. However, few studies have investigated the genetic mechanism for environmental adaptation of *S. maltophilia*.

*S. maltophilia* is also a common bacterium in bile [9]. However, no study has focused on the genome of bile-isolated *S. maltophilia* strains. Here, we report the whole genome sequence of bile-isolated *S. maltophilia* strain ZT1. The bile adaptation and virulence-associated genes of the *S. maltophilia* strain ZT1 were also analyzed.

## Methods

### Strain isolation and characterization

A bile sample of a cholelithiasis patient was obtained for microbe culture and the *S. maltophilia* strain ZT1 was isolated on blood agar. This strain was then cultivated in brain–heart infusion broth for genomic DNA extraction under anaerobic conditions at 37 °C for 12 h. The 16 s rRNA sequence was obtained by PCR using primers 27F (5’-AGAGTTTGATCCTGGCTCAG-3’) and 1492R (5’-GGTTACCTTGTTACGACTT-3’) and then sequenced. The obtained 16 s rRNA sequence was compared to the NCBI website using BLAST.

### Genome sequencing and de novo assembly

A Bacteria DNA isolation kit (OMEGA Bio-Tek Inc., Norcross, GA, USA) was used to extract the genomic DNA from an overnight culture of the *S. maltophilia* strain ZT1. Subsequently, the quality of the genomic DNA was tested using a TBS-380 fluorometer (Turner BioSystems Inc., Sunnyvale, CA, USA). Then, a highly qualified DNA sample (OD260/280 = 1.8– 2.0, total amount > 6 μg) was utilized for genome sequencing. According to the sequencing protocol, approximately 3 μg of genomic DNA was sequenced using an Illumina HiSeq Sequencer (Illumina Inc. San Diego, CA, USA) in the PE150 mode. Illumina pipeline CASAVA v1.8.2 (Illumina Inc. San Diego, CA, USA) was used for base calling to produce the raw sequencing data. Meanwhile, another 3 μg of genomic DNA was used to generate a 20 K template library which was then sequenced using the PacBio RS Platform (Pacific Biosciences of California, Inc., Menlo Park, CA, USA).

The Illumina sequence data were used to establish the complexity of the genome as well as correct the PacBio long reads, as described previously [10]. First, genome assembly was performed using ABySS with multiple-Kmer parameters and then the optimal results was obtained for the assembly [11]. After that, the long reads generated by PacBio were assembled using Canu (https://github.com/marbl/canu). Then, the remaining local inner gaps were filled using GapCloser [12]. Finally, we corrected the single-base polymorphisms for the final assembly results.

Gene annotation was performed using the automatic prokaryotic genome annotation pipeline (https://www.ncbi.nlm.nih.gov/genome/annotation_prok/). The CGView Server was used to create the circular genomic map of strain ZT1 [13]. B, The phylogenetic analysis was performed based on orthologous genes. First, OrthoMCL v2.0 was used to establish orthologous gene families with an E-value of 10^–5^ [14]. Second, the MUSCLE v3.8.31 software was used for multiple alignments [15]. Finally, phylogenetic analyses were performed using PhyML 3.0, based on maximum-likelihood (ML) methods. To calculate the bootstrap values, the GTR + G model was selected for ML analysis using 500 bootstrap replicates [16]. Several strains, including *S. maltophilia* NCTC10257 (LT906480.1), *S. maltophilia* SJTL3(CP029773.1), *S. maltophilia* Sm1 (GCA_001431665.1), *S. maltophilia* Sm2 (GCA_001651505.1), *S. maltophilia* Sm3 (GCA_001068765.1), *S. maltophilia* Sm4a (GCA_001069235.1), *S. maltophilia* Sm4b (GCA_000223885.1), *S. maltophilia* Sm5 (RAVG00000000), *S. maltophilia* Sm6 (GCA_002799015.1), *S. maltophilia* Sm7 (GCA_002798945.1), *S. maltophilia* Sm8 (RATO00000000), *S. maltophilia* Sm9 (GCA_001070945.1), *S. maltophilia* Sm10 (GCA_001297005.1), *S. maltophilia* Sm11 (GCA_002798885.1), *S. maltophilia* Sm12 (RAUV00000000), *S. maltophilia* Sm13 (ERR3300023), *S. maltophilia* Sm14 (GCA_002799245.1), *S. maltophilia* Sm15 (RATP00000000), *S. maltophilia* Sm16 (CP040439.1), *S. maltophilia* Sm17 (RAUD00000000), *S. maltophilia* Sm18 (GCA_002798925.1), *S. maltophilia* Sgn1 (GCA_002025605.1), *S. maltophilia* Sgn2 (GCA_002377295.1), *S. maltophilia* Sgn3 (GCA_000613205.1), *S. maltophilia* Sgn4 (GCA_001676435.1), *S. bentonitica* DSM103927 (GCA_013185915.1), *S. nitritireducens* 2001 (CP016756.1), *S. acidaminiphila* SUNEO (CP019797.1), *S. rhizophila* QL-P4 (CP016294.1); *X. campestris* NEB122 (CP051651.1), *S. indicatrix* DAIF1 (CP037883.1), *S. lactitubi* As-6 (GCA_016921095.1), and *S. tumulicola* JCM 30961 (GCA_014117215.1) were selected for phylogenetic tree analysis. The putative virulence-related genes in the genome of the *S. maltophilia* strain ZT1 were identified using the Virulence Factor Database (VFDB) with a score > 80 and an e-value < 0.001 [17].

## Results and discussion

### General genome features of the *S. maltophilia* strain ZT1

**Fig.1:**
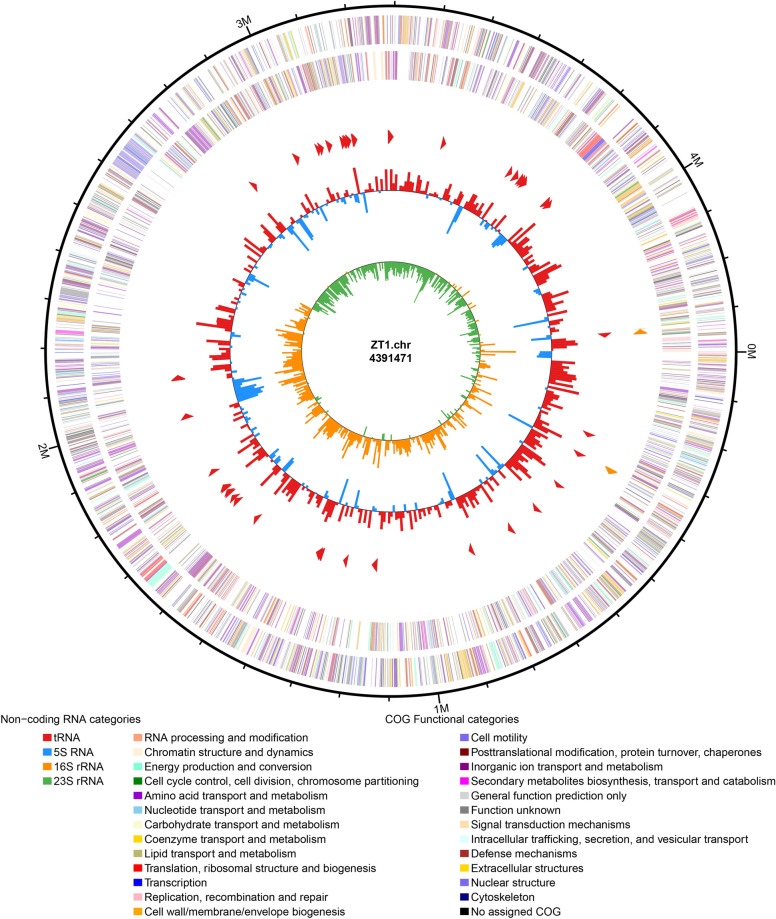
Genome map of *S. maltophilia* ZT1. Circles from the outside to inside showing: (1) DNA coordinates; (2, 3) function-based color-coded mapping of the CDSs predicted on the forward and reverse strands. Functions are color-coding; (4) rRNA genes; (5) tRNA genes; (6) GC plot showing regions above the average (red) and below (blue); (7) GC skew

A total of 2712.2 Mb data containing 18,081,966 raw reads were produced by the Illumina HiSeq Sequencer. After data filtering, 2611.8 Mb data containing 17,458,806 high-quality reads were obtained. Meanwhile, 115,046 (497.3 Mb) high-quality reads were produced by the Pac bio RS Platform with an average read length of 4322 bp. Sequence coverage was > 700-fold. Furthermore, we assembled these reads and obtained a high-quality genome with only one scaffold in this study (Fig. 1). This complete genome is 4.19 Mb with a mean G + C content of 66.51%. After annotation, 3962 protein-coding sequences, seven rRNAs, and 74 tRNA genes were found in the chromosome of strain ZT1.

### Phylogenetic analysis

**Fig.2:**
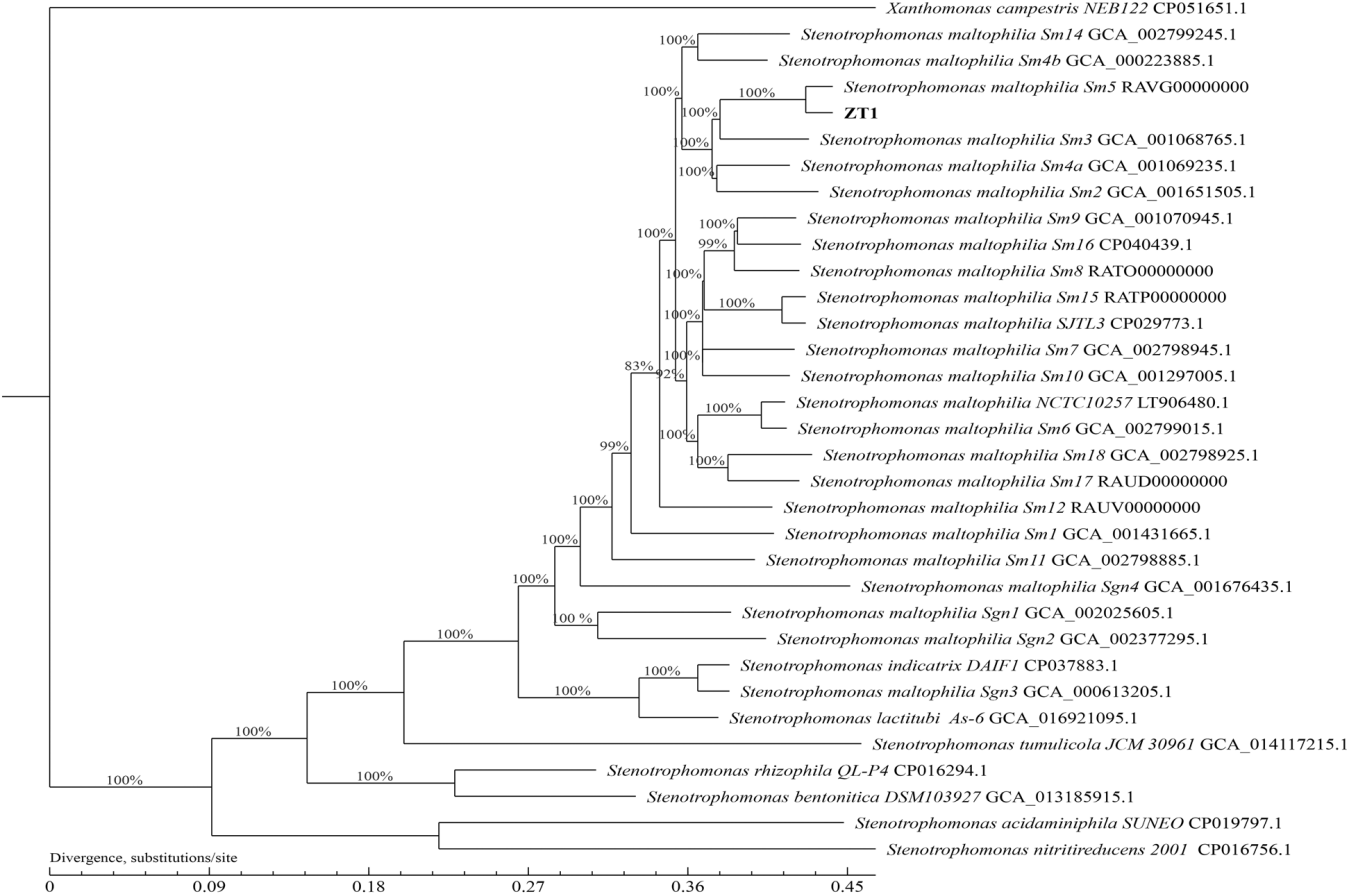
Phylogenetic analysis of *S. maltophilia* ZT1

The 16S rRNA gene sequence of strain ZT1 revealed the taxonomic status of strain ZT1 to be *S. maltophilia* by BLAST(data not shown). Phylogenetic analysis was carried out based on the whole genome DNA-sequence, and the results are shown in Fig. 2. A total of 24 type strains of *S. maltophilia*, including *S. maltophilia* NCTC10257 (LT906480.1), *S. maltophilia* SJTL3(CP029773.1), *S. maltophilia* sm5 (RAVG00000000), were selected as standard. It was found that the strain ZT1 was most closely related to the *S. maltophilia* sm5 clade.

### Identification of genes related to bile adaption

Bile acids are a group of steroids with high stability and biocompatibility. One of the major physiological roles is antimicrobial activity, as they have a structure similar to the antimicrobial peptides [18]. *S. maltophilia* strain ZT1 could live in bile, and which indicates an inherent tolerance to high concentrations of bile. Genomic analysis of the *S. maltophilia* strain ZT1 showed the presence of 22 genes related to bile adaptation (Table 1). Half of them were genes encoding efflux pumps, including emrAB, acrAB, tolC, mexAB, mdtABC, and porin F. We also identified other genes, such as O-antigen biosynthesis-related (rfbABC), DNA binding protein (hupB), DNA mismatch repair protein (mutS), and zinc uptake regulation protein (zur) genes, which are related to bile adaption.

**Table 1:** The genes related to bile adaption in *S. maltophilia* ZT1

Gene ID	Swissprot_tophit_descrip	Swissprot top similarity %	KEGG gene name
ZT1002978	Multidrug export protein EmrB OS = Escherichia coli (strain K12)	49.4	emrB
ZT1002979	Multidrug export protein EmrA OS = Haemophilus influenzae (strain ATCC 51907)	45.2	emrA
ZT1000837	HTH-type transcriptional regulator AcrR OS = Shigella flexneri	31.4	acrR, smeT
ZT1000838	Multidrug efflux pump subunit AcrA OS = Escherichia coli (strain K12)	43.9	acrA, mexA
ZT1000839	Multidrug efflux pump subunit AcrB OS = Escherichia coli (strain K12)	64	acrB, mexB
ZT1000961	Outer membrane protein TolC OS = Vibrio cholerae serotype O1 (strain ATCC 39,315)	31.7	tolC
ZT1001690	Multidrug efflux pump subunit AcrB OS = Escherichia coli (strain K12)	50	acrB, mexB
ZT1001691	Multidrug efflux pump subunit AcrA OS = Escherichia coli (strain K12)	41.2	acrA, mexA
ZT1003728	O-antigen biosynthesis protein RfbC OS = Myxococcus xanthus	42.1	rfbC
ZT1003730	O-antigen export system ATP-binding protein RfbB OS = Myxococcus xanthus	39	ABC-.LPSE.A
ZT1003731	O-antigen export system permease protein RfbA OS = Klebsiella pneumoniae	22	_
ZT1000559	Virulence transcriptional regulatory protein PhoP OS = Salmonella typhi	39.4	_
ZT1000233	Virulence transcriptional regulatory protein PhoP OS = Salmonella typhi	43.7	phoP
ZT1000632	Multidrug resistance protein MdtC OS = Enterobacter sp. (strain 638)	44.5	_
ZT1000633	Multidrug resistance protein MdtB OS = Xenorhabdus bovienii (strain SS-2004)	51.1	mdtB
ZT1000634	Multidrug resistance protein MdtA OS = Dickeya zeae (strain Ech586)	40.3	mdtA
ZT1003430	Outer membrane porin F OS = Pseudomonas aeruginosa (strain ATCC 15,692)	32	TC.OOP
ZT1003393	DNA-binding protein HU OS = Xylella fastidiosa (strain Temecula1 / ATCC 700,964)	76.4	hupB
ZT1002604	UTP–glucose-1-phosphate uridylyltransferase OS = Pseudomonas aeruginosa (strain PAO1)	46.5	galU, galF
ZT1002538	UDP-glucose 4-epimerase OS = Bacillus halodurans (strain ATCC BAA-125)	51.4	galE, GALE
ZT1003099	DNA mismatch repair protein MutS OS = Xanthomonas campestris (strain ATCC 33,913)	89.6	mutS
ZT1003056	Zinc uptake regulation protein OS = Shigella flexneri	37.6	zur

### Analysis of virulence associated genes

*S. maltophilia* is a conditional pathogen and strain ZT1 was isolated from a patient with cholelithiasis. Therefore, analysis of putative virulence-associated genes in the genome of strain ZT1 was performed by aligning gene sequences to VFDB. The results indicated that more than 500 hundred virulence-associated genes were predicted in the genome of strain ZT1 (Additional file 1: Table S1). We found 88 gene related to flagellar biosynthesis in the genome of strain ZT1; additionally, there was a flagellar encoding gene island from ZT1002347 to ZT1002401. The virulence-associated fimbrial assembly protein-encoding genes included pilB, pilQ, pilJ, pilT, pilC, pilU, pilM, pilD, flhA, mrkC, fauA, and flgI. Enterobactin is a virulence factor of *S. maltophilia* [19]. An entCEBFA operon (ZT1002005–ZT1002010) containing genes encoding for proteins of the enterobactin synthesis pathway as well as an enterobactin exporter encoding gene entS (ZT1001564) were found. The efflux pump-associated virulence factors are listed in Additional file 2: Table S2. Several RND family multidrug efflux pumps, NodT family efflux transporter, multidrug ABC transporter and other efflux pumps were found in the genome of strain ZT1, which may be related to antibiotic resistance in *S. maltophilia*. The DNA and/or protein secretion system is another type of virulence factor in *S. maltophilia* [20]. In the genome of strain ZT1, we found two independent type II secretion system operons, one containing 11 genes (xpsDNMLKJIHGFE; ZT1003667–ZT1003677) and the second contained at least another 11 gene (gspGKLMDEFJIHC, ZT1002072–ZT1002085). Some of these were identified as virulence factors, as shown in Additional file 3: Table S3. Meanwhile, a type IV secretion system operon was also found in the genome of strain ZT1, containing genes encoding for virB4, virB8, virB11. These virulence factors might contribute *S. maltophilia* infection and require further investigation.

## Supplementary Information

**Additional file 1. Table S1.** The results indicated that more than 500 hundred virulence-associated genes were predicted in the genome of strain ZT1.

**Additional file 2. Table S2.** The efflux pump associated virulence factor in *S. maltophilia* ZT1 predicted by VFDB.

**Additional file 3. Table S3.** The secretion system associated virulence factor in *S. maltophilia* ZT1 predicted by VFDB.

## Data Availability

The completed genome sequence of *S. maltophilia* strain ZT1 has been deposited into GenBank database with accession number CP071784.
