# Supplementary material for: The complete genome sequence of a bile-isolated Stenotrophomonas maltophilia ZT1
Source: Gut Pathog. 2021 Oct 28;13:64. doi: 10.1186/s13099-021-00456-y (PMC8555292; doi:10.1186/s13099-021-00456-y)
Supplement: Supplementary file 1 — Additional file 1. Table S1. The results indicated that more than 500 hundred virulence-associated genes were predicted in the genome of strain ZT1. [file 13099_2021_456_MOESM1_ESM.docx]

| GeneID | evalue | score | VFid | func |
| --- | --- | --- | --- | --- |
| ZT1000839 | 0.00E+00 | 1265.8 | VFG049146 | (Kvar_3938) hydrophobe/amphiphile efflux-1 (HAE1) family transporter [AcrAB (CVF859)] [Klebsiella variicola At-22] |
| ZT1002043 | 0.00E+00 | 1230.3 | VFG009599(gi:119867330) | (narG) nitrate reductase, alpha subunit [Nitrate reductase (CVF318)] [Mycobacterium sp. KMS] |
| ZT1001154 | 0.00E+00 | 1196 | VFG049190 | (KOX_00005) protein disaggregation chaperone [T6SS-II (CVF861)] [Klebsiella oxytoca KCTC 1686] |
| ZT1002441 | 0.00E+00 | 1190.3 | VFG047717 | (OOM_0012) carbamoyl phosphate synthase large subunit [Pyrimidine biosynthesis (CVF845)] [Francisella noatunensis subsp. orientalis str. Toba 04] |
| ZT1002690 | 0.00E+00 | 1134 | VFG037726(gi:469822480) | (adeG) RND cation/multidrug efflux pump [AdeFGH efflux pump/transport autoinducer (CVF773)] [Acinetobacter baumannii D1279779] |
| ZT1000466 | 6.40E-294 | 1007.7 | VFG049144(gb\|YP_002918165.1) | (acrB) acriflavine resistance protein B [AcrAB (VF0568)] [Klebsiella pneumoniae subsp. pneumoniae NTUH-K2044] |
| ZT1001690 | 3.10E-288 | 988.8 | VFG049146 | (Kvar_3938) hydrophobe/amphiphile efflux-1 (HAE1) family transporter [AcrAB (CVF859)] [Klebsiella variicola At-22] |
| ZT1001131 | 1.90E-271 | 932.2 | VFG042959(gi:28199794) | (pilB) pilus biogenesis protein [type IV pili (AI118)] [Xylella fastidiosa Temecula1] |
| ZT1002150 | 2.00E-270 | 929.1 | VFG044223(gi:104781601) | (bauA) TonB-dependent siderophore receptor BauA [pseudomonine (IA004)] [Pseudomonas entomophila L48] |
| ZT1002458 | 1.90E-266 | 916.4 | VFG036991(gi:218768729) | (mtrD) drug efflux protein [MtrCDE (CVF759)] [Neisseria meningitidis Z2491] |
| ZT1003667 | 9.10E-263 | 903.7 | VFG040901(gi:21230145) | (xpsD) general secretion pathway protein D [xps (SS213)] [Xanthomonas campestris pv. campestris str. ATCC 33913] |
| ZT1003677 | 4.50E-257 | 884.4 | VFG040891(gi:21230135) | (xpsE) general secretion pathway protein E [xps (SS213)] [Xanthomonas campestris pv. campestris str. ATCC 33913] |
| ZT1002143 | 6.70E-247 | 851.3 | VFG018402(gi:161505694) | (mgtB) hypothetical protein [Mg2+ transport (CVF005)] [Salmonella enterica subsp. arizonae serovar 62:z4,z23:-- str. RSK2980] |
| ZT1001223 | 6.60E-240 | 829.3 | VFG019771(gi:218889155) | (chpA) still frameshift putative component of chemotactic signal transduction system [Type IV pili twitching motility related proteins (CVF519)] [Pseudomonas aeruginosa LESB58] |
| ZT1003100 | 6.00E-227 | 784.3 | VFG037032(gi:194099998) | (katA) protein KatA [Catalase (CVF760)] [Neisseria gonorrhoeae NCCP11945] |
| ZT1002096 | 3.20E-221 | 766.1 | VFG037496(gi:213156717) | (AB57_0987) hypothetical protein [Heme utilization (CVF769)] [Acinetobacter baumannii AB0057] |
| ZT1002008 | 2.70E-217 | 753.4 | VFG048490 | (A225_1600) enterobactin synthetase component F [Ent siderophore (CVF849)] [Klebsiella oxytoca E718] |
| ZT1000690 | 9.60E-217 | 750.4 | VFG045692(gi:289163963) | (htpB) molecular chaperone GroEL [Hsp60 (CVF347)] [Legionella longbeachae NSW150] |
| ZT1002393 | 2.70E-216 | 749.2 | VFG014591(gi:152986696) | (flhA) flagellar biosynthesis protein FlhA [Flagella (CVF521)] [Pseudomonas aeruginosa PA7] |
| ZT1001069 | 1.40E-214 | 743.4 | VFG042951(gi:28199566) | (pilQ) fimbrial assembly protein [type IV pili (AI118)] [Xylella fastidiosa Temecula1] |
| ZT1002076 | 1.80E-203 | 706.8 | VFG040877(gi:15595882) | (hxcQ) type II secretion system protein [hxc (SS204)] [Pseudomonas aeruginosa PAO1] |
| ZT1002657 | 3.50E-194 | 675.6 | VFG043573(gi:15605121) | (CT396) molecular chaperone DnaK [MOMP (AI392)] [Chlamydia trachomatis D/UW-3/CX] |
| ZT1003477 | 3.50E-192 | 668.3 | VFG046459 | (Fphi_1039) elongation factor Tu [EF-Tu (CVF827)] [Francisella philomiragia subsp. philomiragia ATCC 25017] |
| ZT1003489 | 3.50E-192 | 668.3 | VFG046459 | (Fphi_1039) elongation factor Tu [EF-Tu (CVF827)] [Francisella philomiragia subsp. philomiragia ATCC 25017] |
| ZT1002077 | 4.80E-188 | 654.8 | VFG040878(gi:15595883) | (hxcR) type II secretion system protein [hxc (SS204)] [Pseudomonas aeruginosa PAO1] |
| ZT1003071 | 1.30E-187 | 654.1 | VFG048542 | (KPK_3197) outer membrane receptor FepA [Salmochelin (CVF850)] [Klebsiella pneumoniae 342] |
| ZT1000191 | 1.00E-173 | 607.1 | VFG022587(gi:333989098) | (icl) isocitrate lyase Icl [Isocitrate lyase (CVF302)] [Mycobacterium sp. JDM601] |
| ZT1001222 | 2.10E-173 | 606.7 | VFG042944(gi:28198752) | (pilJ) pilus biogenesis protein [type IV pili (AI118)] [Xylella fastidiosa Temecula1] |
| ZT1002764 | 1.40E-172 | 604 | VFG020045(gi:218890486) | (plcN) non-hemolytic phospholipase C precursor [Non-hemolytic phospholipase C (CVF541)] [Pseudomonas aeruginosa LESB58] |
| ZT1003659 | 3.90E-172 | 602.8 | VFG042690(gi:283520634) | (mrkC) MrkC [type 3 fimbriae (AI084)] [Citrobacter freundii str. M46] |
| ZT1003328 | 1.00E-171 | 600.1 | VFG042945(gi:28199039) | (pilT) twitching motility protein [type IV pili (AI118)] [Xylella fastidiosa Temecula1] |
| ZT1002044 | 2.60E-171 | 599.4 | VFG009606(gi:41408717) | (narH) NarH [Nitrate reductase (CVF318)] [Mycobacterium avium subsp. paratuberculosis K-10] |
| ZT1001593 | 8.70E-169 | 591.3 | VFG045720(gi:289165297) | (ccmF) cytochrome C-type biogenesis protein CcmF [Cytochrome c muturation (ccm) locus (CVF358)] [Legionella longbeachae NSW150] |
| ZT1001845 | 4.20E-168 | 589.3 | VFG019252(gi:163844264) | (virB4) type IV secretion/conjugal transfer ATPase, VirB4 family [Type IV secretion system (CVF385)] [Brucella suis ATCC 23445] |
| ZT1001128 | 2.00E-166 | 582.8 | VFG042957(gi:77747718) | (pilC) fimbrial assembly protein [type IV pili (AI118)] [Xylella fastidiosa Temecula1] |
| ZT1003327 | 3.30E-163 | 572 | VFG042946(gi:28199040) | (pilU) twitching motility protein [type IV pili (AI118)] [Xylella fastidiosa Temecula1] |
| ZT1001659 | 3.00E-161 | 566.2 | VFG019785(gi:218890653) | (PLES_19131) ORF_10; similar to Asparagine synthase [LPS O-antigen (P. aeruginosa) (CVF520)] [Pseudomonas aeruginosa LESB58] |
| ZT1002535 | 5.90E-161 | 565.5 | VFG007630(gi:37678524) | (wzc) putative tyrosine-protein kinase Wzc [Capsular polysaccharide (CVF282)] [Vibrio vulnificus YJ016] |
| ZT1001679 | 5.20E-160 | 561.6 | VFG037990(gi:523521483) | (BJAB07104_00096) putative UDP-glucose 6-dehydrogenase [Capsule (CVF775)] [Acinetobacter baumannii BJAB07104] |
| ZT1001058 | 2.50E-158 | 557 | VFG042672(gi:206564598) | (cblC) putative outer membrane usher [cable pilus (AI081)] [Burkholderia cenocepacia J2315] |
| ZT1003708 | 6.60E-158 | 554.7 | VFG026293(gi:170732090) | (manC) mannose-1-phosphate guanylyltransferase/mannose-6-phosphate isomerase [Capsule I (CVF645)] [Burkholderia cenocepacia MC0-3] |
| ZT1001210 | 1.20E-155 | 547.7 | VFG010925(gi:148358987) | (relA) GTP pyrophosphokinase ((p)ppGpp synthetase I) stringent stress response RelA [RelA (CVF365)] [Legionella pneumophila str. Corby] |
| ZT1002005 | 3.10E-154 | 542.7 | VFG016132(gi:70730858) | (pchD) 2,3-dihydroxybenzoate-AMP ligase [Pyochelin (CVF553)] [Pseudomonas fluorescens Pf-5] |
| ZT1002484 | 3.90E-153 | 539.7 | VFG031407(gi:433630073) | (ctpV) Putative metal cation transporter P-type ATPase CtpV [Copper exporter (CVF658)] [Mycobacterium canettii CIPT 140070010] |
| ZT1003707 | 4.70E-153 | 538.5 | VFG038167(gi:407930987) | (M3Q_303) hypothetical protein [Capsule (CVF775)] [Acinetobacter baumannii TYTH-1] |
| ZT1002331 | 1.80E-152 | 537.3 | VFG000079(gb\|NP_463763) | (clpC) endopeptidase Clp ATP-binding chain C [ClpC (VF0072)] [Listeria monocytogenes EGD-e] |
| ZT1001016 | 2.90E-152 | 535.8 | VFG013618(gi:113461372) | (hemL) glutamate-1-semialdehyde aminotransferase [Heme biosynthesis (CVF506)] [Haemophilus somnus 129PT] |
| ZT1000294 | 7.00E-149 | 525.4 | VFG014895(gi:116053469) | (algC) phosphomannomutase AlgC [Alginate biosynthesis (CVF522)] [Pseudomonas aeruginosa UCBPP-PA14] |
| ZT1003676 | 1.10E-148 | 523.9 | VFG040892(gi:21230136) | (xpsF) general secretion pathway protein F [xps (SS213)] [Xanthomonas campestris pv. campestris str. ATCC 33913] |
| ZT1002406 | 1.10E-147 | 521.2 | VFG043042(gi:16765263) | (cheA) chemotaxis protein CheA [peritrichous flagella (AI139)] [Salmonella enterica subsp. enterica serovar Typhimurium str. LT2] |
| ZT1001065 | 1.60E-145 | 513.1 | VFG042955(gi:28199570) | (pilM) fimbrial assembly membrane protein [type IV pili (AI118)] [Xylella fastidiosa Temecula1] |
| ZT1002800 | 1.00E-144 | 510.8 | VFG005582(gi:125717729) | (eno) Enolase, putative [Streptococcal enolase (CVF153)] [Streptococcus sanguinis SK36] |
| ZT1002379 | 1.20E-143 | 507.3 | VFG038641(gi:330828802) | (fliI) flagellar protein export ATPase FliI [Polar flagella (CVF786)] [Aeromonas veronii B565] |
| ZT1003794 | 2.00E-142 | 503.8 | VFG031084(gi:433644988) | (zmp1) endothelin-converting enzyme [Zn++ metallophrotease (CVF655)] [Mycobacterium smegmatis JS623] |
| ZT1000114 | 1.60E-140 | 496.9 | VFG009407(gi:145223515) | (glnA1) glutamine synthetase, type I [Glutamine synthesis (CVF311)] [Mycobacterium gilvum PYR-GCK] |
| ZT1003628 | 2.90E-140 | 496.9 | VFG042695(gi:15597326) | (cupA3) usher CupA3 [CupA fimbriae (AI085)] [Pseudomonas aeruginosa PAO1] |
| ZT1001127 | 1.70E-140 | 496.1 | VFG040902(gi:21232531) | (xpsO/pilD) type IV pre-pilin leader peptidase [xps (SS213)] [Xanthomonas campestris pv. campestris str. ATCC 33913] |
| ZT1001103 | 3.10E-139 | 493.8 | VFG037726(gi:469822480) | (adeG) RND cation/multidrug efflux pump [AdeFGH efflux pump/transport autoinducer (CVF773)] [Acinetobacter baumannii D1279779] |
| ZT1001049 | 2.80E-139 | 493.4 | VFG022971(gi:333991010) | (relA) GTP pyrophosphokinase RelA [(p)ppGpp synthesis and hydrolysis (CVF335)] [Mycobacterium sp. JDM601] |
| ZT1003067 | 1.30E-138 | 491.1 | VFG031050(gi:169631610) | (zmp1) Probable zinc metalloprotease [Zn++ metallophrotease (CVF655)] [Mycobacterium abscessus ATCC 19977] |
| ZT1002822 | 1.00E-136 | 484.2 | VFG037908(gi:407930968) | (M3Q_284) UDP-N-acetyl-D-mannosaminuronate dehydrogenase [Capsule (CVF775)] [Acinetobacter baumannii TYTH-1] |
| ZT1002078 | 1.20E-136 | 483.8 | VFG040879(gi:15595884) | (hxcS) type II secretion system protein [hxc (SS204)] [Pseudomonas aeruginosa PAO1] |
| ZT1001424 | 2.00E-134 | 477.2 | VFG031045(gi:108797137) | (zmp1) PgPepO oligopeptidase [Zn++ metallophrotease (CVF655)] [Mycobacterium sp. MCS] |
| ZT1003274 | 1.60E-130 | 464.5 | VFG001731(gi:26249445) | (c3610) bifunctional enterobactin receptor/adhesin protein [IrgA homolog adhesin (Iha) (AI324)] [Escherichia coli CFT073] |
| ZT1000896 | 4.00E-130 | 463 | VFG044124(gi:33593446) | (fauA) ferric alcaligin siderophore receptor [alcaligin (IA010)] [Bordetella pertussis Tohama I] |
| ZT1001458 | 5.70E-130 | 461.8 | VFG013515(gi:148826007) | (mrsA/glmM) predicted phosphomannomutase [Exopolysaccharide (CVF495)] [Haemophilus influenzae PittEE] |
| ZT1002978 | 1.00E-129 | 461.1 | VFG036972(gi:313667722) | (farB) multidrug resistance translocase [FarAB (CVF758)] [Neisseria lactamica 020-06] |
| ZT1001579 | 3.40E-129 | 459.1 | VFG037678(gb\|YP_001847021) | (pgaC) poly-beta-1,6 N-acetyl-D-glucosamine synthase [PNAG (VF0472)] [Acinetobacter baumannii ACICU] |
| ZT1003185 | 1.50E-127 | 454.5 | VFG020085(gi:229590015) | (fpvA) ferripyoverdine receptor precursor [Pyoverdine receptors (CVF552)] [Pseudomonas fluorescens SBW25] |
| ZT1001700 | 1.20E-127 | 454.1 | VFG044382(gi:28192392) | (qbsG) QbsG [thioquinolobactin (IA009)] [Pseudomonas fluorescens ATCC 17400] |
| ZT1002399 | 3.00E-126 | 449.9 | VFG038706(gb\|YP_855924) | (cheA-2) chemotaxis protein CheA [ND (AI144)] [Aeromonas hydrophila subsp. hydrophila ATCC 7966] |
| ZT1003408 | 4.90E-125 | 446 | VFG042931(gi:146329004) | (ppk) polyphosphate kinase [type IV pili (AI117)] [Dichelobacter nodosus VCS1703A] |
| ZT1000444 | 2.30E-125 | 446 | VFG013197(gi:170717931) | (hemB) Porphobilinogen synthase [Heme biosynthesis (CVF506)] [Haemophilus somnus 2336] |
| ZT1002358 | 2.30E-121 | 433 | VFG001242(gb\|NP_249775) | (flgI) flagellar P-ring protein precursor FlgI [Flagella (VF0273)] [Pseudomonas aeruginosa PAO1] |
| ZT1003748 | 6.70E-121 | 432.2 | VFG019785(gi:218890653) | (PLES_19131) ORF_10; similar to Asparagine synthase [LPS O-antigen (P. aeruginosa) (CVF520)] [Pseudomonas aeruginosa LESB58] |
| ZT1003595 | 1.60E-120 | 431.4 | VFG024680(gi:315444326) | (secA2) protein translocase subunit secA [Accessory secretion factor (CVF299)] [Mycobacterium gilvum Spyr1] |
| ZT1001269 | 1.00E-118 | 424.1 | VFG013200(gi:170718716) | (hemE) uroporphyrinogen decarboxylase [Heme biosynthesis (CVF506)] [Haemophilus somnus 2336] |
| ZT1002853 | 1.40E-117 | 421 | VFG013252(gi:33152665) | (msbA) ABC transporter ATP-binding protein MsbA [LOS (CVF494)] [Haemophilus ducreyi 35000HP] |
| ZT1003712 | 1.50E-117 | 420.2 | VFG037923(gi:407930969) | (M3Q_285) nucleoside-diphosphate sugar epimerase [Capsule (CVF775)] [Acinetobacter baumannii TYTH-1] |
| ZT1002372 | 6.90E-113 | 405.2 | VFG007499(gi:27365276) | (flrA) FlaK protein [Flagella (CVF281)] [Vibrio vulnificus CMCP6] |
| ZT1003570 | 1.60E-110 | 397.9 | VFG044104(gi:15599904) | (phuR) heme/hemoglobin uptake outer membrane receptor PhuR precursor [direct heme uptake system (IA049)] [Pseudomonas aeruginosa PAO1] |
| ZT1002636 | 3.50E-110 | 396 | VFG039278(gi:212213222) | (CbuG_1738) trans-2-enoyl-CoA reductase [T4SS effectors (CVF803)] [Coxiella burnetii CbuG_Q212] |
| ZT1001323 | 4.20E-110 | 396 | VFG015769(gi:170720256) | (mucD) protease Do [Alginate regulation (CVF523)] [Pseudomonas putida W619] |
| ZT1002824 | 7.40E-109 | 391.7 | VFG013205(gi:170718942) | (hemN) oxygen-independent coproporphyrinogen III oxidase [Heme biosynthesis (CVF506)] [Haemophilus somnus 2336] |
| ZT1003738 | 7.80E-109 | 391.3 | VFG038473(gi:145298624) | (tapW) pilus retraction ATPase [Tap type IV pili (CVF783)] [Aeromonas salmonicida subsp. salmonicida A449] |
| ZT1001299 | 4.20E-108 | 389.8 | VFG037032(gi:194099998) | (katA) protein KatA [Catalase (CVF760)] [Neisseria gonorrhoeae NCCP11945] |
| ZT1003728 | 1.80E-107 | 387.5 | VFG026307(gi:116688795) | (Bcen2424_0772) glycosyl transferase family protein [Capsule I (CVF645)] [Burkholderia cenocepacia HI2424] |
| ZT1002383 | 1.70E-107 | 386.7 | VFG014510(gi:146307837) | (fliM) flagellar motor switch protein FliM [Flagella (CVF521)] [Pseudomonas mendocina ymp] |
| ZT1003711 | 1.50E-107 | 386.7 | VFG007660(gi:37678485) | (rmlA) D-glucose-1-phosphate thymidylyltransferase [Capsular polysaccharide (CVF282)] [Vibrio vulnificus YJ016] |
| ZT1003670 | 4.30E-107 | 385.6 | VFG040898(gi:21230142) | (xpsL) general secretion pathway protein L [xps (SS213)] [Xanthomonas campestris pv. campestris str. ATCC 33913] |
| ZT1001308 | 1.10E-106 | 384.8 | VFG036828(gi:313667985) | (lptA) sulfatase [Phosphoethanolamine modification (CVF757)] [Neisseria lactamica 020-06] |
| ZT1002739 | 1.70E-106 | 384 | VFG013532(gi:148826097) | (pgi) glucose-6-phosphate isomerase [Exopolysaccharide (CVF495)] [Haemophilus influenzae PittEE] |
| ZT1002016 | 2.60E-106 | 383.6 | VFG036816(gi:194099042) | (lptA) hypothetical protein [Phosphoethanolamine modification (CVF757)] [Neisseria gonorrhoeae NCCP11945] |
| ZT1002605 | 3.00E-105 | 380.2 | VFG001300(gb\|NP_644942) | (cap8D) capsular polysaccharide synthesis enzyme Cap8D [Capsule (VF0003)] [Staphylococcus aureus subsp. aureus MW2] |
| ZT1001963 | 2.40E-104 | 376.7 | VFG042749(gi:15599498) | (tadA) TadA ATPase [type IV pili (AI098)] [Pseudomonas aeruginosa PAO1] |
| ZT1002377 | 2.30E-104 | 376.3 | VFG014429(gi:104782820) | (fliG) flagellar biosynthesis; component of motor switching and energizing FliG [Flagella (CVF521)] [Pseudomonas entomophila L48] |
| ZT1002376 | 2.50E-103 | 373.6 | VFG014418(gi:77457763) | (fliF) flagellar M-ring protein [Flagella (CVF521)] [Pseudomonas fluorescens Pf0-1] |
| ZT1000601 | 1.10E-102 | 371.3 | VFG026700(gi:494692156) | (mbtI) anthranilate synthase component I [Mycobactin (CVF315)] [Mycobacterium tuberculosis str. Haarlem/NITR202] |
| ZT1001031 | 4.50E-102 | 369 | VFG013626(gi:68249065) | (hemN) coproporphyrinogen III oxidase [Heme biosynthesis (CVF506)] [Haemophilus influenzae 86-028NP] |
| ZT1002415 | 1.30E-100 | 364 | VFG025839(gi:206558540) | (cheB) chemotaxis-specific methylesterase [Flagella (CVF643)] [Burkholderia cenocepacia J2315] |
| ZT1000319 | 3.70E-100 | 363.2 | VFG040920(gi:53717647) | (gspE) general secretory pathway protein E [gsp (SS210)] [Burkholderia pseudomallei K96243] |
| ZT1000342 | 2.80E-99 | 359.4 | VFG049099 | (KOX_25105) uridine diphosphate galacturonate 4-epimerase [LPS rfb locus (CVF857)] [Klebsiella oxytoca KCTC 1686] |
| ZT1002635 | 1.40E-98 | 358.2 | VFG042738(gi:15600152) | (fimX) FimX [type IV pili (AI097)] [Pseudomonas aeruginosa PAO1] |
| ZT1001144 | 8.10E-97 | 351.7 | VFG042019(gi:17548952) | (RSp0731) trehalose-6-phosphate synthase [T3SS (SS008)] [Ralstonia solanacearum GMI1000] |
| ZT1003013 | 2.90E-96 | 349.7 | VFG013387(gi:148828224) | (lpxB) lipid-A-disaccharide synthase [LOS (CVF494)] [Haemophilus influenzae PittGG] |
| ZT1000988 | 1.10E-95 | 349 | VFG037498(gi:523530148) | (BJAB0715_01027) Outer membrane receptor protein, mostly Fe transport [Heme utilization (CVF769)] [Acinetobacter baumannii BJAB0715] |
| ZT1002705 | 8.90E-96 | 348.6 | VFG047605 | (FNFX1_1207) hypothetical protein [Cysteine acquisition (CVF840)] [Francisella cf. novicida Fx1] |
| ZT1003599 | 4.70E-96 | 348.6 | VFG013413(gi:68249693) | (lpxC) UDP-3-O-[3-hydroxymyristoyl] N-acetylglucosamine deacetylase [LOS (CVF494)] [Haemophilus influenzae 86-028NP] |
| ZT1002442 | 9.90E-96 | 347.8 | VFG047720 | (Fphi_0805) carbamoyl phosphate synthase small subunit [Pyrimidine biosynthesis (CVF845)] [Francisella philomiragia subsp. philomiragia ATCC 25017] |
| ZT1000633 | 4.80E-95 | 347.1 | VFG037726(gi:469822480) | (adeG) RND cation/multidrug efflux pump [AdeFGH efflux pump/transport autoinducer (CVF773)] [Acinetobacter baumannii D1279779] |
| ZT1002725 | 4.70E-95 | 345.5 | VFG030724(gi:433649467) | (sugC) carbohydrate ABC transporter ATP-binding protein, CUT1 family [Trehalose-recycling ABC transporter (CVF651)] [Mycobacterium smegmatis JS623] |
| ZT1000959 | 2.10E-94 | 343.6 | VFG000141(gb\|NP_253675) | (waaA) lipopolysaccharide core biosynthesis protein WaaP [LPS (VF0085)] [Pseudomonas aeruginosa PAO1] |
| ZT1000802 | 5.60E-93 | 339 | VFG047265 | (OOM_1046) UDP-N-acetylglucosamine pyrophosphorylase/glucosamine-1-phosphate [LPS (CVF834)] [Francisella noatunensis subsp. orientalis str. Toba 04] |
| ZT1003917 | 1.10E-90 | 332 | VFG015348(gi:66047584) | (hopAJ2) type III effector HopAJ2 [P. syringae TTSS effectors (CVF534)] [Pseudomonas syringae pv. syringae B728a] |
| ZT1002660 | 3.10E-90 | 330.1 | VFG037126(gi:385328078) | (recN) putative DNA repair protein [Recombinational repair protein (CVF763)] [Neisseria meningitidis alpha710] |
| ZT1003008 | 2.80E-89 | 326.6 | VFG019924(gi:218890126) | (mucP) putative membrane-associated zinc metalloprotease [Alginate regulation (CVF523)] [Pseudomonas aeruginosa LESB58] |
| ZT1002354 | 3.30E-89 | 326.2 | VFG014198(gi:146307869) | (flgE) flagellar basal body FlaE domain protein [Flagella (CVF521)] [Pseudomonas mendocina ymp] |
| ZT1003306 | 6.40E-89 | 325.5 | VFG042736(gi:15599658) | (rpoN) RNA polymerase factor sigma-54 [type IV pili (AI097)] [Pseudomonas aeruginosa PAO1] |
| ZT1000695 | 7.20E-88 | 321.6 | VFG044185(gi:37676958) | (VVA1298) phospho-2-dehydro-3-deoxyheptonate aldolase [vulnibactin (IA023)] [Vibrio vulnificus YJ016] |
| ZT1002538 | 2.00E-87 | 320.1 | VFG016307(gi:30023295) | (BC5263) UDP-glucose 4-epimerase [Polysaccharide capsule (CVF567)] [Bacillus cereus ATCC 14579] |
| ZT1000632 | 8.00E-87 | 319.7 | VFG049144(gb\|YP_002918165.1) | (acrB) acriflavine resistance protein B [AcrAB (VF0568)] [Klebsiella pneumoniae subsp. pneumoniae NTUH-K2044] |
| ZT1002996 | 3.00E-86 | 317.4 | VFG044448(gi:15599847) | (cupE5) hypothetical protein [CupE fimbriae (AI449)] [Pseudomonas aeruginosa PAO1] |
| ZT1000476 | 2.30E-86 | 316.6 | VFG047506 | (Fphi_1799) biotin synthase [Biotin metabolism (CVF838)] [Francisella philomiragia subsp. philomiragia ATCC 25017] |
| ZT1002869 | 8.10E-86 | 316.6 | VFG042949(gi:28199488) | (pilY1) fimbrial assembly protein [type IV pili (AI118)] [Xylella fastidiosa Temecula1] |
| ZT1000840 | 6.50E-86 | 315.5 | VFG034657(gi:215485614) | (ibeB) copper/silver efflux system outer membrane protein CusC [Invasion of brain endothelial cells (Ibes) (CVF429)] [Escherichia coli O127:H6 str. E2348/69] |
| ZT1002356 | 8.40E-86 | 314.3 | VFG014222(gi:70729006) | (flgG) flagellar basal-body rod protein FlgG [Flagella (CVF521)] [Pseudomonas fluorescens Pf-5] |
| ZT1002395 | 2.10E-85 | 313.2 | VFG014624(gi:104782802) | (fleN) flagellar number regulator FleN [Flagella (CVF521)] [Pseudomonas entomophila L48] |
| ZT1000772 | 6.40E-85 | 312.8 | VFG044334(gb\|YP_002920266) | (iroN) salmochelin receptor IroN [Sal (VF0563)] [Klebsiella pneumoniae subsp. pneumoniae NTUH-K2044] |
| ZT1001134 | 8.50E-85 | 312 | VFG014002(gi:146283959) | (pilS) two-component sensor PilS [Type IV pili biosynthesis (CVF518)] [Pseudomonas stutzeri A1501] |
| ZT1003671 | 4.60E-85 | 312 | VFG040897(gi:21230141) | (xpsK) general secretion pathway protein K [xps (SS213)] [Xanthomonas campestris pv. campestris str. ATCC 33913] |
| ZT1002343 | 4.30E-84 | 310.5 | VFG043139(gi:28901366) | (scrC) sensory box/GGDEF family protein SrcC [lateral flagella (AI142)] [Vibrio parahaemolyticus RIMD 2210633] |
| ZT1000426 | 3.10E-84 | 309.7 | VFG041881(gi:34498047) | (cpbD) carbohydrate-binding protein [Cpi-2 encoded T3SS (SPI-2 like) (SS023)] [Chromobacterium violaceum ATCC 12472] |
| ZT1002389 | 1.30E-83 | 308.5 | VFG043139(gi:28901366) | (scrC) sensory box/GGDEF family protein SrcC [lateral flagella (AI142)] [Vibrio parahaemolyticus RIMD 2210633] |
| ZT1000577 | 8.20E-84 | 308.1 | VFG016502(gi:47459418) | (pdhB) pyruvate dehydrogenase E1 component beta subunit [PDH-B (CVF588)] [Mycoplasma mobile 163K] |
| ZT1002803 | 1.40E-83 | 307 | VFG011414(gb\|NP_539767) | (kdsA) 2-dehydro-3-deoxyphosphooctonate aldolase [LPS (CVF383)] [Brucella melitensis bv. 1 str. 16M] |
| ZT1001522 | 6.80E-83 | 305.8 | VFG029777(gi:169631512) | (fadE5) Probable acyl-CoA dehydrogenase FadE [GPL locus (CVF650)] [Mycobacterium abscessus ATCC 19977] |
| ZT1003122 | 2.30E-82 | 304.3 | VFG043139(gi:28901366) | (scrC) sensory box/GGDEF family protein SrcC [lateral flagella (AI142)] [Vibrio parahaemolyticus RIMD 2210633] |
| ZT1002370 | 4.50E-82 | 302.8 | VFG042736(gi:15599658) | (rpoN) RNA polymerase factor sigma-54 [type IV pili (AI097)] [Pseudomonas aeruginosa PAO1] |
| ZT1000465 | 5.00E-82 | 302.4 | VFG049135 | (Kvar_3937) RND family efflux transporter MFP subunit [AcrAB (CVF859)] [Klebsiella variicola At-22] |
| ZT1001978 | 1.30E-80 | 298.5 | VFG044177(gi:28901511) | (pvuA) ferric vibrioferrin receptor [vibrioferrin (IA038)] [Vibrio parahaemolyticus RIMD 2210633] |
| ZT1001715 | 7.90E-81 | 298.1 | VFG044377(gi:28192387) | (qbsB) QbsB [thioquinolobactin (IA009)] [Pseudomonas fluorescens ATCC 17400] |
| ZT1001353 | 1.70E-80 | 297.7 | VFG037212(gi:169634285) | (plcD) phospholipase D [Phospholipase D (CVF767)] [Acinetobacter baumannii SDF] |
| ZT1002475 | 1.80E-80 | 297.4 | VFG000313(gb\|NP_207965) | (gluP) glucose/galactose transporter [LPS (VF0056)] [Helicobacter pylori 26695] |
| ZT1000122 | 2.20E-79 | 293.9 | VFG015681(gi:170722859) | (fleR) two component, sigma54 specific, transcriptional regulator, Fis family [Flagella (CVF521)] [Pseudomonas putida W619] |
| ZT1002360 | 6.30E-79 | 292.7 | VFG001244(gb\|NP_249777) | (flgK) flagellar hook-associated protein 1 FlgK [Flagella (VF0273)] [Pseudomonas aeruginosa PAO1] |
| ZT1001471 | 9.60E-79 | 292.4 | VFG017492(gi:119867644) | (nuoG) NADH-quinone oxidoreductase, chain G [NuoG (CVF620)] [Mycobacterium sp. KMS] |
| ZT1000732 | 2.40E-78 | 290.8 | VFG026971(gi:494697073) | (sigA/rpoV) RNA polymerase sigma factor [Sigma A (CVF325)] [Mycobacterium tuberculosis CAS/NITR204] |
| ZT1000617 | 2.80E-78 | 290.8 | VFG015078(gi:146306761) | (gacS) multi-sensor hybrid histidine kinase [GacS/GacA two-component system (CVF529)] [Pseudomonas mendocina ymp] |
| ZT1001132 | 1.30E-78 | 290.4 | VFG013993(gi:146283960) | (pilR) two-component response regulator PilR [Type IV pili biosynthesis (CVF518)] [Pseudomonas stutzeri A1501] |
| ZT1001089 | 2.80E-78 | 289.7 | VFG005360(gi:24378857) | (plr/gapA) glyceraldehyde-3-phosphate dehydrogenase [Streptococcal plasmin receptor/GAPDH (CVF123)] [Streptococcus mutans UA159] |
| ZT1002099 | 7.00E-78 | 288.9 | VFG037513(gi:184157197) | (ACICU_00877) hypothetical protein [Heme utilization (CVF769)] [Acinetobacter baumannii ACICU] |
| ZT1001836 | 1.80E-77 | 287.7 | VFG040415(gi:379019260) | (rvhD4) type IV secretion system protein VirB8 [Rvh T4SS (CVF804)] [Rickettsia rickettsii Hlp#2] |
| ZT1000596 | 2.10E-77 | 286.6 | VFG007811(gi:523912069) | (purC) phosphoribosylaminoimidazole-succinocarboxamide synthase [Purine synthesis (CVF306)] [Mycobacterium yongonense 05-1390] |
| ZT1002527 | 4.80E-77 | 286.6 | VFG007643(gi:59710763) | (wbfB) WbfB protein [Capsular polysaccharide (CVF282)] [Vibrio fischeri ES114] |
| ZT1001067 | 2.10E-77 | 286.2 | VFG042953(gi:28199568) | (pilO) fimbrial assembly membrane protein [type IV pili (AI118)] [Xylella fastidiosa Temecula1] |
| ZT1002644 | 1.20E-76 | 285.8 | VFG043139(gi:28901366) | (scrC) sensory box/GGDEF family protein SrcC [lateral flagella (AI142)] [Vibrio parahaemolyticus RIMD 2210633] |
| ZT1002350 | 5.00E-77 | 285.4 | VFG038818(gi:330830308) | (cheV) chemotaxis protein methyltransferase CheV [Polar flagella (CVF786)] [Aeromonas veronii B565] |
| ZT1000777 | 6.30E-77 | 285 | VFG013198(gi:170718229) | (hemC) porphobilinogen deaminase [Heme biosynthesis (CVF506)] [Haemophilus somnus 2336] |
| ZT1000838 | 1.40E-76 | 284.3 | VFG049129 | (KPHS_11890) acridine efflux pump [AcrAB (CVF859)] [Klebsiella pneumoniae subsp. pneumoniae HS11286] |
| ZT1003500 | 2.00E-76 | 283.9 | VFG013607(gi:113460953) | (hemA) glutamyl-tRNA reductase [Heme biosynthesis (CVF506)] [Haemophilus somnus 129PT] |
| ZT1000805 | 1.80E-75 | 280.8 | VFG014390(gi:104782823) | (fleR) response regulator FleR [Flagella (CVF521)] [Pseudomonas entomophila L48] |
| ZT1003783 | 1.90E-75 | 280 | VFG025942(gi:206558532) | (motA) flagellar motor protein MotA [Flagella (CVF643)] [Burkholderia cenocepacia J2315] |
| ZT1001841 | 6.40E-75 | 278.5 | VFG019266(gi:163844257) | (virB11) P-type DNA transfer ATPase VirB11 [Type IV secretion system (CVF385)] [Brucella suis ATCC 23445] |
| ZT1000425 | 7.60E-75 | 278.5 | VFG041881(gi:34498047) | (cpbD) carbohydrate-binding protein [Cpi-2 encoded T3SS (SPI-2 like) (SS023)] [Chromobacterium violaceum ATCC 12472] |
| ZT1002537 | 2.30E-74 | 276.9 | VFG007665(gi:15640950) | (cpsA) capsular polysaccharide biosynthesis glycosyltransferase, putative [Capsular polysaccharide (CVF282)] [Vibrio cholerae O1 biovar El Tor str. N16961] |
| ZT1002362 | 3.00E-74 | 276.6 | VFG001854(gb\|YP_095369) | (fliC) flagellin [Flagella (VF0157)] [Legionella pneumophila subsp. pneumophila str. Philadelphia 1] |
| ZT1002392 | 6.20E-74 | 275.4 | VFG014578(gi:152983829) | (flhB) flagellar biosynthetic protein FlhB [Flagella (CVF521)] [Pseudomonas aeruginosa PA7] |
| ZT1002413 | 6.30E-74 | 275 | VFG025865(gi:206558538) | (cheR) chemotaxis protein methyltransferase [Flagella (CVF643)] [Burkholderia cenocepacia J2315] |
| ZT1001554 | 1.50E-73 | 275 | VFG043139(gi:28901366) | (scrC) sensory box/GGDEF family protein SrcC [lateral flagella (AI142)] [Vibrio parahaemolyticus RIMD 2210633] |
| ZT1002648 | 2.80E-73 | 273.9 | VFG013252(gi:33152665) | (msbA) ABC transporter ATP-binding protein MsbA [LOS (CVF494)] [Haemophilus ducreyi 35000HP] |
| ZT1003009 | 1.40E-72 | 271.9 | VFG016675(gi:121602491) | (omp89) surface antigen/outer membrane protein, OMP85 family [Omp89 (CVF601)] [Bartonella bacilliformis KC583] |
| ZT1000160 | 1.50E-72 | 271.6 | VFG011124(gi:115422956) | (bvgS) virulence sensor protein [Two-component system (CVF377)] [Bordetella avium 197N] |
| ZT1002449 | 1.90E-72 | 271.2 | VFG045721(gi:289163734) | (feoB) ferrous iron transport protein B [Ferrous iron transport (CVF359)] [Legionella longbeachae NSW150] |
| ZT1003360 | 1.70E-72 | 270 | VFG038840(gi:507521851) | (flmH) 3-oxoacyl-ACP reductase [Polar flagella (VF0473)] [Aeromonas hydrophila ML09-119] |
| ZT1000955 | 5.70E-72 | 268.9 | VFG037781(gi:523529590) | (lpsB) Glycosyltransferase [LPS (CVF774)] [Acinetobacter baumannii BJAB0715] |
| ZT1000598 | 1.00E-71 | 267.3 | VFG046612 | (FN3523_1292) Ribulose-phosphate 3-epimerase [Capsule (CVF833)] [Francisella cf. tularensis subsp. novicida 3523] |
| ZT1003291 | 2.10E-71 | 266.9 | VFG047569 | (FNFX1_0412) hypothetical protein [Purine (CVF839)] [Francisella cf. novicida Fx1] |
| ZT1002400 | 2.50E-71 | 266.2 | VFG015731(gi:170722825) | (motC) MotA/TolQ/ExbB proton channel [Flagella (CVF521)] [Pseudomonas putida W619] |
| ZT1003358 | 6.10E-70 | 262.3 | VFG009135(gi:383308045) | (kasB) 3-oxoacyl-(acyl carrier protein) synthase II [FAS-II (CVF300)] [Mycobacterium tuberculosis RGTB327] |
| ZT1003730 | 1.00E-69 | 261.5 | VFG049050 | (KPN_02485) O-antigen export - NBD component [LPS rfb locus (CVF857)] [Klebsiella pneumoniae subsp. pneumoniae MGH 78578] |
| ZT1003447 | 2.00E-69 | 260.8 | VFG015510(gi:152985851) | (phzC1) phenazine biosynthesis protein PhzC [Phenazines biosynthesis (CVF536)] [Pseudomonas aeruginosa PA7] |
| ZT1001249 | 1.40E-69 | 260.8 | VFG012939(gi:110804318) | (gtrB) bactoprenol glucosyl transferase [LPS glucosylation (CVF473)] [Shigella flexneri 5 str. 8401] |
| ZT1002619 | 6.50E-69 | 260.4 | VFG037726(gi:469822480) | (adeG) RND cation/multidrug efflux pump [AdeFGH efflux pump/transport autoinducer (CVF773)] [Acinetobacter baumannii D1279779] |
| ZT1000275 | 2.60E-69 | 260.4 | VFG019921(gi:218893537) | (algW) AlgW protein [Alginate regulation (CVF523)] [Pseudomonas aeruginosa LESB58] |
| ZT1003301 | 2.40E-69 | 260 | VFG017759(gi:153951764) | (kpsF) arabinose-5-phosphate isomerase [Capsule biosynthesis and transport (CVF393)] [Campylobacter jejuni subsp. doylei 269.97] |
| ZT1003623 | 2.00E-68 | 258.5 | VFG035982(gi:260866483) | (ehaA) AidA-I adhesin-like protein [EhaA, AIDA-I type (CVF743)] [Escherichia coli O111:H- str. 11128] |
| ZT1003784 | 6.50E-69 | 258.5 | VFG014690(gi:70734060) | (motB) flagellar motor protein [Flagella (CVF521)] [Pseudomonas fluorescens Pf-5] |
| ZT1002364 | 1.10E-68 | 258.1 | VFG014302(gi:146307857) | (fliC) flagellin domain protein [Flagella (CVF521)] [Pseudomonas mendocina ymp] |
| ZT1001221 | 8.20E-69 | 257.3 | VFG042943(gi:28198751) | (pilI) pilus biogenesis protein [type IV pili (AI118)] [Xylella fastidiosa Temecula1] |
| ZT1003709 | 2.40E-68 | 256.5 | VFG048811 | (A225_3879) dTDP-4-dehydrorhamnose reductase [Capsule (CVF854)] [Klebsiella oxytoca E718] |
| ZT1001173 | 4.30E-68 | 255.4 | VFG039536(gb\|NP_820549) | (CBU_1566) Coxiella Dot/Icm type IVB secretion system translocated effector [T4SS effectors (CVF803)] [Coxiella burnetii RSA 493] |
| ZT1002363 | 7.00E-68 | 255.4 | VFG001854(gb\|YP_095369) | (fliC) flagellin [Flagella (VF0157)] [Legionella pneumophila subsp. pneumophila str. Philadelphia 1] |
| ZT1003384 | 1.00E-67 | 254.6 | VFG013278(gi:148827418) | (opsX/rfaC) ADP-heptose--lipooligosaccharide heptosyltransferase I [LOS (CVF494)] [Haemophilus influenzae PittGG] |
| ZT1002424 | 2.20E-67 | 254.2 | VFG030400(gi:333989012) | (fadD13) fatty-acid--CoA ligase [MymA operon (CVF649)] [Mycobacterium sp. JDM601] |
| ZT1000467 | 2.40E-67 | 253.8 | VFG037010(gb\|NP_274717) | (mtrE) multidrug efflux pump channel protein MtrE [MtrCDE (VF0451)] [Neisseria meningitidis MC58] |
| ZT1001227 | 2.20E-67 | 253.8 | VFG047522 | (bioA) adenosylmethionine-8-amino-7-oxononanoate aminotransferase [Biotin metabolism (CVF838)] [Francisella novicida U112] |
| ZT1001580 | 4.20E-67 | 253.4 | VFG037664(gb\|YP_001847022) | (pgaB) poly-beta-1,6-N-acetyl-D-glucosamine N-deacetylase PgaB [PNAG (VF0472)] [Acinetobacter baumannii ACICU] |
| ZT1003396 | 1.80E-67 | 253.1 | VFG000077(gb\|NP_465991) | (clpP) ATP-dependent Clp protease proteolytic subunit [ClpP (VF0074)] [Listeria monocytogenes EGD-e] |
| ZT1002593 | 3.50E-67 | 253.1 | VFG044389(gi:6959513) | (pdtorfF) putative sulfurylase [Pyridine-2,6-dithiocarboxylic acid (PDTC) (IA030)] [Pseudomonas stutzeri KC] |
| ZT1003749 | 3.40E-67 | 252.3 | VFG038219(gb\|YP_001845364) | (bfmR) biofilm-controlling response regulator [BfmRS (VF0463)] [Acinetobacter baumannii ACICU] |
| ZT1002176 | 1.40E-66 | 251.1 | VFG013990(gi:70732614) | (pilR) sigma-54 dependent DNA-binding response regulator PilR, putative [Type IV pili biosynthesis (CVF518)] [Pseudomonas fluorescens Pf-5] |
| ZT1000976 | 3.40E-66 | 250.4 | VFG047610 | (F7308_1193) Gamma-glutamyltranspeptidase [Cysteine acquisition (CVF840)] [Francisella sp. TX077308] |
| ZT1002524 | 6.10E-66 | 248.8 | VFG048944 | (KOX_25205) polysaccharide export protein Wza [Capsule (CVF854)] [Klebsiella oxytoca KCTC 1686] |
| ZT1002979 | 6.50E-66 | 248.8 | VFG036942(gi:194099567) | (farA) Efflux pump protein, fatty acid resistance [FarAB (CVF758)] [Neisseria gonorrhoeae NCCP11945] |
| ZT1000312 | 1.50E-65 | 247.3 | VFG013615(gi:68249709) | (hemH) ferrochelatase [Heme biosynthesis (CVF506)] [Haemophilus influenzae 86-028NP] |
| ZT1000074 | 6.50E-65 | 246.5 | VFG011124(gi:115422956) | (bvgS) virulence sensor protein [Two-component system (CVF377)] [Bordetella avium 197N] |
| ZT1002851 | 3.60E-65 | 245.7 | VFG038845(gi:507522470) | (nueA) 3-deoxy-D-manno-octulosonate cytidylyltransferase [Polar flagella (VF0473)] [Aeromonas hydrophila ML09-119] |
| ZT1001965 | 1.80E-64 | 244.2 | VFG042751(gi:15599500) | (rcpA) RcpA [type IV pili (AI098)] [Pseudomonas aeruginosa PAO1] |
| ZT1002312 | 2.30E-64 | 243.4 | VFG044249(gi:145300222) | (hutC) ABC-type hemin transporter, permease protein [direct heme uptake system (IA062)] [Aeromonas salmonicida subsp. salmonicida A449] |
| ZT1001350 | 1.90E-63 | 241.9 | VFG011125(gi:33591460) | (sphB1) autotransporter subtilisin-like protease [SphB1 (SS058)] [Bordetella pertussis Tohama I] |
| ZT1001068 | 1.80E-63 | 239.6 | VFG042952(gi:28199567) | (pilP) fimbrial assembly protein [type IV pili (AI118)] [Xylella fastidiosa Temecula1] |
| ZT1003669 | 5.30E-63 | 238.4 | VFG040899(gi:21230143) | (xpsM) general secretion pathway protein M [xps (SS213)] [Xanthomonas campestris pv. campestris str. ATCC 33913] |
| ZT1001789 | 2.40E-62 | 237.7 | VFG033884(gi:386641996) | (hlyB) hemolysin secretion protein HlyB [Alpha-hemolysin (CVF453)] [Escherichia coli ABU 83972] |
| ZT1001321 | 8.70E-62 | 234.2 | VFG014917(gi:146306499) | (algU) RNA polymerase, sigma-24 subunit, ECF subfamily [Alginate regulation (CVF523)] [Pseudomonas mendocina ymp] |
| ZT1003793 | 9.40E-61 | 233 | VFG045467(gb\|YP_107884) | (cdpA) cyclic di-GMP phosphodiesterase [CdpA (VF0432)] [Burkholderia pseudomallei K96243] |
| ZT1002852 | 3.20E-61 | 233 | VFG013243(gi:68248610) | (lpxK) tetraacyldisaccharide 4'-kinase [LOS (CVF494)] [Haemophilus influenzae 86-028NP] |
| ZT1001116 | 7.60E-61 | 232.6 | VFG042349(gi:296112646) | (MCR_0419) Moraxella catarrhalis adherence protein McaP [McaP (SS111)] [Moraxella catarrhalis RH4] |
| ZT1000579 | 3.60E-61 | 232.6 | VFG044381(gi:28192391) | (qbsF) QbsF [thioquinolobactin (IA009)] [Pseudomonas fluorescens ATCC 17400] |
| ZT1002396 | 5.20E-61 | 231.9 | VFG014632(gi:28869183) | (fliA) flagellar biosynthesis sigma factor FliA [Flagella (CVF521)] [Pseudomonas syringae pv. tomato str. DC3000] |
| ZT1002386 | 9.30E-61 | 231.1 | VFG025426(gi:206561840) | (fliP) flagellar biosynthesis protein FliP [Flagella (CVF643)] [Burkholderia cenocepacia J2315] |
| ZT1003030 | 2.90E-60 | 230.7 | VFG038918(gb\|YP_855893) | (rtxE) RTX toxin transporter, ATPase protein [The repeat in toxin (RTX) (CVF795)] [Aeromonas hydrophila subsp. hydrophila ATCC 7966] |
| ZT1001066 | 3.10E-60 | 229.6 | VFG042954(gi:28199569) | (pilN) fimbrial assembly membrane protein [type IV pili (AI118)] [Xylella fastidiosa Temecula1] |
| ZT1002042 | 2.40E-59 | 227.3 | VFG022816(gi:169630604) | (narK2) integral membrane nitrite extrusion protein NarK3 [Nitrate/nitrite transporter (CVF320)] [Mycobacterium abscessus ATCC 19977] |
| ZT1002604 | 1.80E-59 | 226.9 | VFG016382(gi:30023307) | (BC5275) UTP--glucose-1-phosphate uridylyltransferase [Polysaccharide capsule (CVF567)] [Bacillus cereus ATCC 14579] |
| ZT1000919 | 4.30E-59 | 226.1 | VFG030291(gi:126437598) | (adhD) alcohol dehydrogenase [MymA operon (CVF649)] [Mycobacterium sp. JLS] |
| ZT1002004 | 1.00E-58 | 224.9 | VFG044366(gi:307131896) | (cbsC) isochorismate hydroxymutase 2, chrysobactin biosynthesis [chrysobactin (IA012)] [Dickeya dadantii 3937] |
| ZT1001691 | 1.10E-58 | 224.9 | VFG036978(gi:194099138) | (mtrC) antibiotic resistance efflux pump component [MtrCDE (CVF759)] [Neisseria gonorrhoeae NCCP11945] |
| ZT1001304 | 6.60E-58 | 222.2 | VFG044123(gi:33593445) | (alcS) putative drug resistance translocase [alcaligin (IA010)] [Bordetella pertussis Tohama I] |
| ZT1000304 | 4.40E-58 | 222.2 | VFG042739(gi:15600525) | (crc) catabolite repression control protein [type IV pili (AI097)] [Pseudomonas aeruginosa PAO1] |
| ZT1001157 | 9.10E-58 | 221.1 | VFG038219(gb\|YP_001845364) | (bfmR) biofilm-controlling response regulator [BfmRS (VF0463)] [Acinetobacter baumannii ACICU] |
| ZT1002880 | 7.60E-58 | 221.1 | VFG001867(gb\|YP_096960) | (sodB) superoxide dismutase [SodB (VF0169)] [Legionella pneumophila subsp. pneumophila str. Philadelphia 1] |
| ZT1002401 | 1.60E-57 | 220.7 | VFG014731(gi:146307821) | (motD) OmpA/MotB domain protein [Flagella (CVF521)] [Pseudomonas mendocina ymp] |
| ZT1002741 | 1.80E-57 | 220.3 | VFG009301(gi:118467890) | (panC) pantoate--beta-alanine ligase [Pantothenate synthesis (CVF305)] [Mycobacterium smegmatis str. MC2 155] |
| ZT1003710 | 2.70E-57 | 219.2 | VFG007663(gi:37678487) | (rmlC) dTDP-6-deoxy-D-xylo-4-hexulose-3,5-epimerase [Capsular polysaccharide (CVF282)] [Vibrio vulnificus YJ016] |
| ZT1002394 | 1.30E-56 | 218.4 | VFG038688(gi:145298361) | (flhF) flagellar biosynthesis protein FlhF [Polar flagella (CVF786)] [Aeromonas salmonicida subsp. salmonicida A449] |
| ZT1000604 | 8.20E-57 | 218.4 | VFG022640(gi:183983227) | (trpD) anthranilate phosphoribosyltransferase [Tryptophan synthesis (CVF308)] [Mycobacterium marinum M] |
| ZT1000224 | 2.10E-56 | 217.2 | VFG029181(gi:494691572) | (fadE14) acyl-CoA dehydrogenase FADE13 [Mycobactin (CVF315)] [Mycobacterium tuberculosis str. Haarlem/NITR202] |
| ZT1001219 | 6.30E-57 | 217.2 | VFG042942(gi:77747668) | (pilG) pilus protein [type IV pili (AI118)] [Xylella fastidiosa Temecula1] |
| ZT1001547 | 5.00E-56 | 215.3 | VFG038840(gi:507521851) | (flmH) 3-oxoacyl-ACP reductase [Polar flagella (VF0473)] [Aeromonas hydrophila ML09-119] |
| ZT1002911 | 7.70E-56 | 215.3 | VFG030700(gi:507420313) | (sugC) sugar ABC transporter ATP-binding protein SugC [Trehalose-recycling ABC transporter (CVF651)] [Mycobacterium abscessus subsp. bolletii 50594] |
| ZT1000655 | 1.10E-55 | 214.9 | VFG047592 | (F7308_0433) phosphoribosylaminoimidazole-succinocarboxamide synthase / phosphoribosylamine--glycine ligase [Purine (CVF839)] [Francisella sp. TX077308] |
| ZT1002306 | 3.40E-55 | 214.2 | VFG042670(gi:206564596) | (cblS) two-component regulatory system sensor kinase protein [cable pilus (AI081)] [Burkholderia cenocepacia J2315] |
| ZT1001507 | 1.40E-55 | 213.8 | VFG011805(gi:118475485) | (CFF8240_1412) aspartate racemase [LOS (CVF396)] [Campylobacter fetus subsp. fetus 82-40] |
| ZT1003718 | 1.90E-55 | 213.4 | VFG038841(gi:145297258) | (flmH) 3-oxoacyl-ACP reductase [Polar flagella (CVF786)] [Aeromonas salmonicida subsp. salmonicida A449] |
| ZT1003005 | 2.50E-55 | 213 | VFG045688(gi:383329042) | (uppS) undecaprenyl diphosphate synthase [Capsule (CVF618)] [Enterococcus faecium Aus0004] |
| ZT1002691 | 1.20E-54 | 211.5 | VFG037714(gi:169633158) | (adeF) multidrug ABC transporter [AdeFGH efflux pump/transport autoinducer (CVF773)] [Acinetobacter baumannii SDF] |
| ZT1001687 | 2.50E-54 | 210.3 | VFG044398(gi:6959522) | (pdtorfO) putative acyl-CoA dehydrogenase [Pyridine-2,6-dithiocarboxylic acid (PDTC) (IA030)] [Pseudomonas stutzeri KC] |
| ZT1002402 | 2.90E-54 | 209.5 | VFG038724(gi:145298370) | (ASA_1361) SOJ-like and chromosome partitioning protein [Polar flagella (CVF786)] [Aeromonas salmonicida subsp. salmonicida A449] |
| ZT1001510 | 3.80E-54 | 208.8 | VFG000077(gb\|NP_465991) | (clpP) ATP-dependent Clp protease proteolytic subunit [ClpP (VF0074)] [Listeria monocytogenes EGD-e] |
| ZT1001252 | 4.00E-54 | 208.8 | VFG016234(gi:16079238) | (hlyIII) hypothetical protein [Hemolysin III (CVF560)] [Bacillus subtilis subsp. subtilis str. 168] |
| ZT1003769 | 1.00E-53 | 208.4 | VFG009141(gi:433648643) | (kasB) 3-oxoacyl-(acyl-carrier-protein) synthase [FAS-II (CVF300)] [Mycobacterium smegmatis JS623] |
| ZT1002006 | 6.80E-54 | 208 | VFG048414 | (entB) isochorismatase [Ent siderophore (CVF849)] [Klebsiella pneumoniae 342] |
| ZT1001278 | 1.70E-53 | 208 | VFG041031(gi:15597555) | (sfa3) transcriptional regulator [HSI-3 (SS180)] [Pseudomonas aeruginosa PAO1] |
| ZT1003675 | 4.80E-54 | 208 | VFG040893(gi:21230137) | (xpsG) general secretion pathway protein G [xps (SS213)] [Xanthomonas campestris pv. campestris str. ATCC 33913] |
| ZT1001264 | 5.00E-53 | 207.6 | VFG011124(gi:115422956) | (bvgS) virulence sensor protein [Two-component system (CVF377)] [Bordetella avium 197N] |
| ZT1000346 | 4.50E-53 | 207.6 | VFG037008(gi:313667899) | (mtrD) drug efflux protein [MtrCDE (CVF759)] [Neisseria lactamica 020-06] |
| ZT1000631 | 5.00E-53 | 206.8 | VFG044284(gi:197284142) | (pbtB) TonB-dependent siderophore receptor [Proteobactin (IA034)] [Proteus mirabilis HI4320] |
| ZT1002311 | 4.10E-53 | 205.7 | VFG044250(gi:145300223) | (hutD) ABC-type hemin transporter, ATP-binding protein [direct heme uptake system (IA062)] [Aeromonas salmonicida subsp. salmonicida A449] |
| ZT1002009 | 4.10E-53 | 205.7 | VFG019337(gi:225685881) | (dhbA) probable 2,3-dihydro-2,3-dihydroxybenzoate dehydrogenase protein [Brucebactin (CVF386)] [Brucella melitensis ATCC 23457] |
| ZT1001289 | 4.60E-53 | 204.5 | VFG042950(gi:28199509) | (pilH) PilH family regulatory protein [type IV pili (AI118)] [Xylella fastidiosa Temecula1] |
| ZT1000965 | 8.40E-52 | 203.4 | VFG036992(gb\|NP_274718) | (mtrD) multiple transferable resistance system protein MtrD [MtrCDE (VF0451)] [Neisseria meningitidis MC58] |
| ZT1003357 | 6.20E-52 | 202.6 | VFG026700(gi:494692156) | (mbtI) anthranilate synthase component I [Mycobactin (CVF315)] [Mycobacterium tuberculosis str. Haarlem/NITR202] |
| ZT1002732 | 2.90E-52 | 202.6 | VFG015785(gi:170722540) | (gacA) two component transcriptional regulator, LuxR family [GacS/GacA two-component system (CVF529)] [Pseudomonas putida W619] |
| ZT1000775 | 6.20E-52 | 202.2 | VFG014977(gi:152988551) | (algZ) alginate biosynthesis protein AlgZ/FimS [Alginate regulation (CVF523)] [Pseudomonas aeruginosa PA7] |
| ZT1000395 | 2.10E-51 | 200.7 | VFG009135(gi:383308045) | (kasB) 3-oxoacyl-(acyl carrier protein) synthase II [FAS-II (CVF300)] [Mycobacterium tuberculosis RGTB327] |
| ZT1003414 | 2.90E-51 | 199.5 | VFG013323(gi:148826622) | (lpxH) UDP-2,3-diacylglucosamine hydrolase [LOS (CVF494)] [Haemophilus influenzae PittEE] |
| ZT1002426 | 1.10E-50 | 199.5 | VFG019942(gi:229591212) | (gacS) hybrid sensory histidine kinase in two-component regulatory system with UvrY [GacS/GacA two-component system (CVF529)] [Pseudomonas fluorescens SBW25] |
| ZT1001032 | 3.90E-51 | 198.7 | VFG013269(gi:33152420) | (orfM) putative deoxyribonucleotide triphosphate pyrophosphatase [LOS (CVF494)] [Haemophilus ducreyi 35000HP] |
| ZT1003805 | 1.10E-50 | 198.4 | VFG029755(gi:118470803) | (ecf) extra cytoplasmic sigma factor [GPL locus (CVF650)] [Mycobacterium smegmatis str. MC2 155] |
| ZT1003672 | 5.40E-51 | 198.4 | VFG040896(gi:21230140) | (xpsJ) general secretion pathway protein J [xps (SS213)] [Xanthomonas campestris pv. campestris str. ATCC 33913] |
| ZT1000776 | 1.10E-50 | 197.6 | VFG014964(gi:152989087) | (algR) alginate biosynthesis regulatory protein AlgR [Alginate regulation (CVF523)] [Pseudomonas aeruginosa PA7] |
| ZT1001639 | 4.50E-50 | 196.8 | VFG013251(gi:148827214) | (msbA) lipid A export ATP-binding protein MsbA [LOS (CVF494)] [Haemophilus influenzae PittGG] |
| ZT1002618 | 7.70E-50 | 196.8 | VFG049136 | (A225_1326) RND efflux system [AcrAB (CVF859)] [Klebsiella oxytoca E718] |
| ZT1000299 | 5.10E-50 | 196.8 | VFG031423(gi:523914198) | (ctpV) P-ATPase superfamily P-type ATPase copper transporter [Copper exporter (CVF658)] [Mycobacterium yongonense 05-1390] |
| ZT1002397 | 1.30E-50 | 196.4 | VFG007572(gi:37680647) | (cheY) chemotaxis protein CheY [Flagella (CVF281)] [Vibrio vulnificus YJ016] |
| ZT1001596 | 1.60E-49 | 193.7 | VFG001856(gb\|YP_094893) | (ccmC) cytochrome c-type biogenesis protein CcmC, putative heme lyase for CcmE [CcmC (VF0292)] [Legionella pneumophila subsp. pneumophila str. Philadelphia 1] |
| ZT1003752 | 2.60E-49 | 193 | VFG031738(gi:169631126) | (regX3) Sensory transduction protein RegX3 [RegX3 (CVF667)] [Mycobacterium abscessus ATCC 19977] |
| ZT1001627 | 1.10E-48 | 190.7 | VFG001867(gb\|YP_096960) | (sodB) superoxide dismutase [SodB (VF0169)] [Legionella pneumophila subsp. pneumophila str. Philadelphia 1] |
| ZT1001265 | 6.30E-48 | 190.7 | VFG042670(gi:206564596) | (cblS) two-component regulatory system sensor kinase protein [cable pilus (AI081)] [Burkholderia cenocepacia J2315] |
| ZT1003406 | 1.60E-48 | 190.3 | VFG031730(gi:118470709) | (regX3) DNA-binding response regulator RegX3 [RegX3 (CVF667)] [Mycobacterium smegmatis str. MC2 155] |
| ZT1003083 | 5.60E-48 | 189.5 | VFG016050(gi:66045186) | (pvdH) diaminobutyrate--2-oxoglutarate aminotransferase [Pyoverdine (CVF551)] [Pseudomonas syringae pv. syringae B728a] |
| ZT1002409 | 2.50E-48 | 189.1 | VFG002530(gb\|YP_109901) | (cheW) chemotaxis protein CheW [Flagella (VF0430)] [Burkholderia pseudomallei K96243] |
| ZT1001924 | 5.00E-48 | 189.1 | VFG044122(gi:33593444) | (alcR) transcriptional regulator [alcaligin (IA010)] [Bordetella pertussis Tohama I] |
| ZT1002812 | 6.80E-48 | 189.1 | VFG012163(gi:18309419) | (hlyB) probable hemolysin [Hemolysin (CVF417)] [Clostridium perfringens str. 13] |
| ZT1000233 | 6.00E-48 | 188.3 | VFG004061(gi:16760106) | (phoP) transcriptional regulatory protein PhoP, regulator of virulence determinants [PhoPQ (CVF010)] [Salmonella enterica subsp. enterica serovar Typhi str. CT18] |
| ZT1002072 | 6.60E-48 | 187.6 | VFG040873(gi:15595878) | (hxcT) HxcT pseudopilin [hxc (SS204)] [Pseudomonas aeruginosa PAO1] |
| ZT1001156 | 2.50E-47 | 187.2 | VFG038238(gi:523529861) | (bfmS) Signal transduction histidine kinase [Two-component system (CVF778)] [Acinetobacter baumannii BJAB0715] |
| ZT1000198 | 2.30E-47 | 187.2 | VFG029181(gi:494691572) | (fadE14) acyl-CoA dehydrogenase FADE13 [Mycobactin (CVF315)] [Mycobacterium tuberculosis str. Haarlem/NITR202] |
| ZT1001013 | 7.00E-47 | 185.7 | VFG044347(gi:307130370) | (acsF) 4-aminobutyrate aminotransferase [achromobactin (IA011)] [Dickeya dadantii 3937] |
| ZT1000961 | 1.30E-46 | 184.9 | VFG044140(gi:218927856) | (hasF) outer membrane channel protein [HasA-type hemophore-mediated heme uptake system (IA044)] [Yersinia pestis CO92] |
| ZT1003353 | 4.50E-47 | 184.5 | VFG042947(gi:28199377) | (pilZ) type IV fimbriae assembly protein [type IV pili (AI118)] [Xylella fastidiosa Temecula1] |
| ZT1000762 | 5.70E-46 | 184.1 | VFG042333(gi:197286187) | (PMI2341) autotransporter [Proteus toxic agglutinin (Pta) (SS112)] [Proteus mirabilis HI4320] |
| ZT1003733 | 2.30E-46 | 184.1 | VFG044286(gi:197284144) | (pbtD) pyridoxal-phosphate dependent enzyme [Proteobactin (IA034)] [Proteus mirabilis HI4320] |
| ZT1002951 | 1.70E-46 | 183.7 | VFG038219(gb\|YP_001845364) | (bfmR) biofilm-controlling response regulator [BfmRS (VF0463)] [Acinetobacter baumannii ACICU] |
| ZT1002968 | 1.70E-46 | 183.7 | VFG038219(gb\|YP_001845364) | (bfmR) biofilm-controlling response regulator [BfmRS (VF0463)] [Acinetobacter baumannii ACICU] |
| ZT1003199 | 1.60E-46 | 183.7 | VFG038219(gb\|YP_001845364) | (bfmR) biofilm-controlling response regulator [BfmRS (VF0463)] [Acinetobacter baumannii ACICU] |
| ZT1002688 | 4.10E-46 | 183.3 | VFG037738(gi:523524047) | (adeH) Outer membrane protein [AdeFGH efflux pump/transport autoinducer (CVF773)] [Acinetobacter baumannii BJAB07104] |
| ZT1002459 | 3.20E-46 | 183.3 | VFG036978(gi:194099138) | (mtrC) antibiotic resistance efflux pump component [MtrCDE (CVF759)] [Neisseria gonorrhoeae NCCP11945] |
| ZT1003953 | 3.50E-46 | 183.3 | VFG015989(gi:26990905) | (pvdM) dipeptidase, putative [Pyoverdine (CVF551)] [Pseudomonas putida KT2440] |
| ZT1002518 | 1.10E-45 | 183 | VFG015782(gi:167032264) | (gacS) multi-sensor hybrid histidine kinase [GacS/GacA two-component system (CVF529)] [Pseudomonas putida GB-1] |
| ZT1003775 | 1.30E-45 | 182.2 | VFG011124(gi:115422956) | (bvgS) virulence sensor protein [Two-component system (CVF377)] [Bordetella avium 197N] |
| ZT1000394 | 5.90E-46 | 181.8 | VFG038840(gi:507521851) | (flmH) 3-oxoacyl-ACP reductase [Polar flagella (VF0473)] [Aeromonas hydrophila ML09-119] |
| ZT1002898 | 7.70E-46 | 181.8 | VFG007910(gi:118466584) | (ddrA) daunorubicin resistance ATP-binding protein DrrA [PDIM (phthiocerol dimycocerosate) and PGL (phenolic glycolipid) biosynthesis and transport (CVF288)] [Mycobacterium avium 104] |
| ZT1001915 | 1.00E-45 | 181.4 | VFG044083(gb\|NP_250948) | (ptxR) transcriptional regulator PtxR [pyoverdine (IA001)] [Pseudomonas aeruginosa PAO1] |
| ZT1003632 | 7.20E-45 | 180.3 | VFG015073(gi:26988382) | (gacS) sensor histidine kinase/response regulator GacS [GacS/GacA two-component system (CVF529)] [Pseudomonas putida KT2440] |
| ZT1001530 | 2.20E-45 | 180.3 | VFG016039(gi:152983967) | (pvdJ) thermophilic carboxylesterase Est2 [Pyoverdine (CVF551)] [Pseudomonas aeruginosa PA7] |
| ZT1003062 | 2.30E-45 | 179.9 | VFG031731(gi:126433265) | (regX3) two component transcriptional regulator [RegX3 (CVF667)] [Mycobacterium sp. JLS] |
| ZT1002359 | 5.00E-45 | 179.5 | VFG014264(gi:146281776) | (flgJ) flagellar protein FlgJ [Flagella (CVF521)] [Pseudomonas stutzeri A1501] |
| ZT1002357 | 3.70E-45 | 179.1 | VFG014227(gi:152984579) | (flgH) flagellar L-ring protein precursor FlgH [Flagella (CVF521)] [Pseudomonas aeruginosa PA7] |
| ZT1002414 | 4.20E-45 | 178.7 | VFG025852(gi:206558539) | (cheD) chemoreceptor glutamine deamidase CheD [Flagella (CVF643)] [Burkholderia cenocepacia J2315] |
| ZT1002355 | 6.60E-45 | 178.3 | VFG003434(gi:51596012) | (flgF) flagellar basal-body rod protein FlgF [Flagella (cluster I) (CVF039)] [Yersinia pseudotuberculosis IP 32953] |
| ZT1003076 | 8.20E-45 | 177.9 | VFG041842(gi:34498090) | (armR) two-component response regulator [Cpi-1a + Cpi-1 (SPI-1 like) (SS016)] [Chromobacterium violaceum ATCC 12472] |
| ZT1003660 | 1.50E-44 | 177.2 | VFG042536(gb\|AAA92619) | (f17d-D) F17 fimbrial chaperone [F17 pili (AI037)] [Escherichia coli str. 111KH86] |
| ZT1001536 | 1.10E-43 | 175.6 | VFG016011(gi:26990907) | (pvdO) hypothetical protein [Pyoverdine (CVF551)] [Pseudomonas putida KT2440] |
| ZT1003426 | 1.60E-43 | 174.5 | VFG047489 | (Fphi_1800) 8-amino-7-oxononanoate synthase [Biotin metabolism (CVF838)] [Francisella philomiragia subsp. philomiragia ATCC 25017] |
| ZT1003564 | 9.40E-44 | 174.5 | VFG009866(gi:126437246) | (mprA) two component transcriptional regulator, winged helix family [MprA/B (CVF333)] [Mycobacterium sp. JLS] |
| ZT1002110 | 1.20E-43 | 174.1 | VFG041842(gi:34498090) | (armR) two-component response regulator [Cpi-1a + Cpi-1 (SPI-1 like) (SS016)] [Chromobacterium violaceum ATCC 12472] |
| ZT1003723 | 3.30E-43 | 173.3 | VFG011157(gi:33599142) | (bplF) lipopolysaccharide biosynthesis protein [LPS (CVF380)] [Bordetella bronchiseptica RB50] |
| ZT1000608 | 1.90E-43 | 172.9 | VFG042734(gi:15595849) | (vfr) cAMP-regulatory protein [type IV pili (AI097)] [Pseudomonas aeruginosa PAO1] |
| ZT1001827 | 1.00E-42 | 172.6 | VFG016094(gi:152984528) | (fpvA) ferrichrome-iron receptor (Ferric hydroxamate uptake)(Ferric hydroxamate receptor) [Pyoverdine receptors (CVF552)] [Pseudomonas aeruginosa PA7] |
| ZT1001998 | 4.10E-43 | 172.2 | VFG001867(gb\|YP_096960) | (sodB) superoxide dismutase [SodB (VF0169)] [Legionella pneumophila subsp. pneumophila str. Philadelphia 1] |
| ZT1003040 | 2.00E-42 | 171.8 | VFG045467(gb\|YP_107884) | (cdpA) cyclic di-GMP phosphodiesterase [CdpA (VF0432)] [Burkholderia pseudomallei K96243] |
| ZT1000203 | 7.50E-43 | 171.4 | VFG039615(gb\|NP_820749) | (coxH3) Coxiella Dot/Icm type IVB secretion system translocated effector [Dot/Icm (SS051)] [Coxiella burnetii RSA 493] |
| ZT1000939 | 1.80E-42 | 170.2 | VFG038840(gi:507521851) | (flmH) 3-oxoacyl-ACP reductase [Polar flagella (VF0473)] [Aeromonas hydrophila ML09-119] |
| ZT1000796 | 3.50E-42 | 170.2 | VFG016960(gi:15834665) | (cdsN) type III secretion system ATPase [Type III secretion system (CVF619)] [Chlamydia muridarum Nigg] |
| ZT1002987 | 7.20E-42 | 169.5 | VFG011198(gi:33594855) | (wbmI) putative asparagine synthetase [LPS (CVF380)] [Bordetella parapertussis 12822] |
| ZT1000559 | 4.80E-42 | 168.7 | VFG004061(gi:16760106) | (phoP) transcriptional regulatory protein PhoP, regulator of virulence determinants [PhoPQ (CVF010)] [Salmonella enterica subsp. enterica serovar Typhi str. CT18] |
| ZT1003674 | 4.70E-42 | 168.3 | VFG040894(gi:21230138) | (xpsH) general secretion pathway protein H [xps (SS213)] [Xanthomonas campestris pv. campestris str. ATCC 33913] |
| ZT1003731 | 9.70E-42 | 167.9 | VFG049061 | (KPN_02486) O-antigen export - TMD component [LPS rfb locus (CVF857)] [Klebsiella pneumoniae subsp. pneumoniae MGH 78578] |
| ZT1001586 | 6.30E-41 | 166.4 | VFG016186(gi:28869794) | (ybtP) ABC transporter, ATP-binding/permease protein [yersiniabactin (IA005)] [Pseudomonas syringae pv. tomato str. DC3000] |
| ZT1001098 | 7.70E-41 | 165.2 | VFG044254(gi:151220272) | (sbnA) O-Acetyl serine sulfhydrylase [staphyloferrin B (IA027)] [Staphylococcus aureus subsp. aureus str. Newman] |
| ZT1001614 | 9.20E-41 | 164.9 | VFG030565(gi:31793499) | (sugA) sugar-transport integral membrane protein ABC transporter UspA [Trehalose-recycling ABC transporter (CVF651)] [Mycobacterium bovis AF2122/97] |
| ZT1000463 | 7.20E-41 | 164.9 | VFG031730(gi:118470709) | (regX3) DNA-binding response regulator RegX3 [RegX3 (CVF667)] [Mycobacterium smegmatis str. MC2 155] |
| ZT1001682 | 2.20E-40 | 164.9 | VFG014101(gi:15598353) | (PA3157) probable acetyltransferase [LPS O-antigen (P. aeruginosa) (CVF520)] [Pseudomonas aeruginosa PAO1] |
| ZT1000296 | 9.80E-41 | 164.5 | VFG031730(gi:118470709) | (regX3) DNA-binding response regulator RegX3 [RegX3 (CVF667)] [Mycobacterium smegmatis str. MC2 155] |
| ZT1000153 | 6.70E-40 | 163.3 | VFG037365(gi:387123166) | (bauA) TonB-dependent siderophore receptor [Acinetobactin (CVF768)] [Acinetobacter baumannii MDR-TJ] |
| ZT1000423 | 3.90E-40 | 162.9 | VFG007909(gi:41407336) | (ddrA) DrrA [PDIM (phthiocerol dimycocerosate) and PGL (phenolic glycolipid) biosynthesis and transport (CVF288)] [Mycobacterium avium subsp. paratuberculosis K-10] |
| ZT1003330 | 4.30E-40 | 162.5 | VFG022639(gi:333989135) | (proC) pyrroline-5-carboxylate reductase ProC [Proline synthesis (CVF307)] [Mycobacterium sp. JDM601] |
| ZT1002345 | 9.20E-40 | 162.5 | VFG043641(gi:28898151) | (scrG) hypothetical protein [lateral flagella (AI142)] [Vibrio parahaemolyticus RIMD 2210633] |
| ZT1000942 | 8.00E-40 | 161.4 | VFG041842(gi:34498090) | (armR) two-component response regulator [Cpi-1a + Cpi-1 (SPI-1 like) (SS016)] [Chromobacterium violaceum ATCC 12472] |
| ZT1002214 | 1.60E-39 | 160.6 | VFG030724(gi:433649467) | (sugC) carbohydrate ABC transporter ATP-binding protein, CUT1 family [Trehalose-recycling ABC transporter (CVF651)] [Mycobacterium smegmatis JS623] |
| ZT1003407 | 4.50E-39 | 159.8 | VFG031699(gi:507423001) | (senX3) two-component histidine kinase SenX3 [SenX3 (CVF666)] [Mycobacterium abscessus subsp. bolletii 50594] |
| ZT1002365 | 4.80E-39 | 159.8 | VFG014323(gi:26991065) | (fliD) flagellar cap protein FliD [Flagella (CVF521)] [Pseudomonas putida KT2440] |
| ZT1000794 | 8.90E-39 | 159.1 | VFG041940(gi:21230693) | (hrcN) type III secretion system ATPase [T3SS (SS009)] [Xanthomonas campestris pv. campestris str. ATCC 33913] |
| ZT1003890 | 4.30E-39 | 158.3 | VFG039549(gb\|NP_820577) | (CBU_1594) Coxiella Dot/Icm type IVB secretion system translocated effector [T4SS effectors (CVF803)] [Coxiella burnetii RSA 493] |
| ZT1003347 | 4.60E-38 | 157.1 | VFG044341(gi:292489704) | (foxR) ferrioxamine E receptor [desferrioxamine (IA033)] [Erwinia amylovora CFBP1430] |
| ZT1000429 | 1.60E-38 | 157.1 | VFG007914(gi:120404101) | (ddrA) daunorubicin resistance ABC transporter ATPase subunit [PDIM (phthiocerol dimycocerosate) and PGL (phenolic glycolipid) biosynthesis and transport (CVF288)] [Mycobacterium vanbaalenii PYR-1] |
| ZT1001123 | 1.50E-38 | 157.1 | VFG027205(gi:507420010) | (mprA) two component response transcriptional regulatory protein MprA [MprA/B (CVF333)] [Mycobacterium abscessus subsp. bolletii 50594] |
| ZT1000683 | 1.20E-37 | 156 | VFG043209(gb\|YP_001006778) | (cheD) methyl-accepting chemotaxis protein CheD [peritrichous flagella (AI145)] [Yersinia enterocolitica subsp. enterocolitica 8081] |
| ZT1003668 | 6.50E-38 | 155.2 | VFG040900(gi:21230144) | (xpsN) general secretion pathway protein N [xps (SS213)] [Xanthomonas campestris pv. campestris str. ATCC 33913] |
| ZT1003630 | 1.70E-37 | 153.7 | VFG010955(gi:33597568) | (fimB) chaperone protein [Fimbriae (CVF368)] [Bordetella parapertussis 12822] |
| ZT1000868 | 2.20E-37 | 153.7 | VFG048845 | (KOX_25145) 6-phosphogluconate dehydrogenase [Capsule (CVF854)] [Klebsiella oxytoca KCTC 1686] |
| ZT1003167 | 1.50E-37 | 153.7 | VFG024200(gi:406033401) | (devR/dosR) two-component system response regulator [DevR/S (CVF334)] [Mycobacterium indicus pranii MTCC 9506] |
| ZT1002557 | 4.20E-37 | 153.7 | VFG006259(gi:59800668) | (fbpC) ABC transporter, ATP-binding protein, iron related [ABC transporter (CVF197)] [Neisseria gonorrhoeae FA 1090] |
| ZT1000886 | 3.40E-37 | 153.3 | VFG045531(gb\|YP_095688) | (lpg1661) Dot/Icm type IV secretion system effector [Dot/Icm (VF0156)] [Legionella pneumophila subsp. pneumophila str. Philadelphia 1] |
| ZT1002606 | 3.10E-37 | 153.3 | VFG014113(gi:15598341) | (wbpL) glycosyltransferase WbpL [LPS O-antigen (P. aeruginosa) (CVF520)] [Pseudomonas aeruginosa PAO1] |
| ZT1002924 | 9.50E-37 | 152.1 | VFG047534 | (FNFX1_0825) hypothetical protein [Biotin metabolism (CVF838)] [Francisella cf. novicida Fx1] |
| ZT1001311 | 8.60E-37 | 151.4 | VFG024189(gi:387874359) | (mprA) two component response transcriptional regulatory protein MprA [MprA/B (CVF333)] [Mycobacterium intracellulare str. MOTT36Y] |
| ZT1002388 | 1.20E-36 | 151 | VFG014575(gi:146307832) | (fliR) flagellar biosynthetic protein FliR [Flagella (CVF521)] [Pseudomonas mendocina ymp] |
| ZT1002053 | 1.40E-36 | 150.6 | VFG036559(gi:313668880) | (fbpC) iron-uptake permease ATP-binding protein [ABC transporter (CVF197)] [Neisseria lactamica 020-06] |
| ZT1003416 | 3.90E-36 | 150.2 | VFG045809 | (EC958_0639) hypothetical protein [SCI-I T6SS (CVF734)] [Escherichia coli O25b:H4-ST131] |
| ZT1002858 | 1.80E-36 | 150.2 | VFG042127(gi:13475293) | (ttsI) two-component response regulator [T3SS (SS026)] [Mesorhizobium loti MAFF303099] |
| ZT1001587 | 4.50E-36 | 150.2 | VFG044228(gi:104781606) | (PSEEN2497) ABC transporter permease/ATP-binding protein [pseudomonine (IA004)] [Pseudomonas entomophila L48] |
| ZT1001971 | 2.30E-35 | 149.4 | VFG018685(gi:163867448) | (badA/vomp/brp) surface protein/Bartonella adhesin [Bartonella adhesin A (CVF599)] [Bartonella tribocorum CIP 105476] |
| ZT1001842 | 7.60E-36 | 148.7 | VFG011545(gi:23499836) | (virB1) type IV secretion system protein VirB1 [Type IV secretion system (CVF385)] [Brucella suis 1330] |
| ZT1002048 | 1.00E-35 | 148.7 | VFG009654(gi:119867331) | (narK2) major facilitator superfamily MFS_1 [Nitrate/nitrite transporter (CVF320)] [Mycobacterium sp. KMS] |
| ZT1000332 | 3.40E-35 | 147.5 | VFG030387(gi:433645508) | (tgs4) acyl-CoA synthetase (AMP-forming)/AMP-acid ligase II [MymA operon (CVF649)] [Mycobacterium smegmatis JS623] |
| ZT1002905 | 2.60E-35 | 146.7 | VFG012163(gi:18309419) | (hlyB) probable hemolysin [Hemolysin (CVF417)] [Clostridium perfringens str. 13] |
| ZT1003386 | 2.10E-35 | 146.7 | VFG019990(gi:229593376) | (pppA) putative phosphatase [Hcp secretion island-1 encoded type VI secretion system (H-T6SS) (CVF535)] [Pseudomonas fluorescens SBW25] |
| ZT1002708 | 2.60E-35 | 146 | VFG000320(gb\|NP_208266) | (kdtB) lipopolysaccharide core biosynthesis protein [LPS (VF0056)] [Helicobacter pylori 26695] |
| ZT1000730 | 4.60E-35 | 146 | VFG037756(gi:169632698) | (lpxM) lipid A biosynthesis lauroyl acyltransferase [LPS (CVF774)] [Acinetobacter baumannii SDF] |
| ZT1003339 | 6.30E-35 | 145.6 | VFG044083(gb\|NP_250948) | (ptxR) transcriptional regulator PtxR [pyoverdine (IA001)] [Pseudomonas aeruginosa PAO1] |
| ZT1002361 | 1.80E-34 | 144.4 | VFG011316(gi:115422778) | (flgL) flagellar hook-associated protein 3 [Flagella (CVF382)] [Bordetella avium 197N] |
| ZT1002315 | 1.50E-34 | 144.4 | VFG048537 | (KPN_01678) putative oxidoreductase, Fe-S subunit [Salmochelin (CVF850)] [Klebsiella pneumoniae subsp. pneumoniae MGH 78578] |
| ZT1002019 | 9.90E-35 | 144.4 | VFG009863(gi:118473019) | (mprA) DNA-binding response regulator [MprA/B (CVF333)] [Mycobacterium smegmatis str. MC2 155] |
| ZT1001220 | 9.00E-35 | 143.7 | VFG014026(gi:77461528) | (pilH) Response regulator receiver domain protein (CheY) [Type IV pili twitching motility related proteins (CVF519)] [Pseudomonas fluorescens Pf0-1] |
| ZT1000163 | 5.00E-34 | 142.9 | VFG029172(gi:397679176) | (fadE14) acyl-CoA dehydrogenase YngJ [Mycobactin (CVF315)] [Mycobacterium abscessus subsp. bolletii str. GO 06] |
| ZT1002531 | 3.60E-34 | 142.5 | VFG001344(gb\|NP_688176) | (cpsM) polysaccharide biosynthesis protein CpsM(V) [Capsule (VF0274)] [Streptococcus agalactiae 2603V/R] |
| ZT1002460 | 4.00E-34 | 142.5 | VFG011626(gi:17988319) | (bvrR) Transcriptional regulatory protein BvrR [BvrR-BvrS (VF0368)] [Brucella melitensis bv. 1 str. 16M] |
| ZT1002353 | 5.00E-34 | 142.1 | VFG038802(gi:330830304) | (flgD) flagellar hook capping protein [Polar flagella (CVF786)] [Aeromonas veronii B565] |
| ZT1003750 | 2.20E-33 | 141 | VFG038245(gi:407931632) | (bfmS) signal transduction histidine kinase [Two-component system (CVF778)] [Acinetobacter baumannii TYTH-1] |
| ZT1001678 | 1.60E-33 | 141 | VFG010532(gi:54293773) | (mip) macrophage infectivity potentiator [Mip (CVF349)] [Legionella pneumophila str. Lens] |
| ZT1001976 | 1.50E-33 | 140.6 | VFG004044(gi:16762553) | (mgtC) conserved hyopthetical protein [Mg2+ transport (CVF005)] [Salmonella enterica subsp. enterica serovar Typhi str. CT18] |
| ZT1002663 | 1.10E-33 | 140.2 | VFG000478(gi:16764063) | (fur) transcriptional repressor of iron-responsive genes (Fur family) (ferric uptake regulator) [Fur (VF0113)] [Salmonella enterica subsp. enterica serovar Typhimurium str. LT2] |
| ZT1000969 | 2.10E-33 | 139.8 | VFG023954(gi:315445135) | (leuD) 3-isopropylmalate dehydratase small subunit [Leucine synthesis (CVF309)] [Mycobacterium gilvum Spyr1] |
| ZT1000948 | 9.50E-33 | 139.8 | VFG018826(gi:162420651) | (YpAngola_A3944) putative sensor histidine kinase/response regulator EsrA [TTSS (chromosomally encoded) (CVF046)] [Yersinia pestis Angola] |
| ZT1000261 | 4.20E-33 | 139 | VFG030673(gi:15827534) | (sugC) ABC transporter ATP-binding protein [Trehalose-recycling ABC transporter (CVF651)] [Mycobacterium leprae TN] |
| ZT1001920 | 9.50E-33 | 138.3 | VFG044083(gb\|NP_250948) | (ptxR) transcriptional regulator PtxR [pyoverdine (IA001)] [Pseudomonas aeruginosa PAO1] |
| ZT1000725 | 1.10E-32 | 137.9 | VFG013319(gi:33152306) | (lgtF) UDP-glucose--Lipooligosaccharide beta 1-4 glucosyltransferase [LOS (CVF494)] [Haemophilus ducreyi 35000HP] |
| ZT1003442 | 8.80E-33 | 137.5 | VFG041304(gb\|YP_095978) | (lirB) Dot/Icm type IV secretion system effector LirB [Dot/Icm (SS047)] [Legionella pneumophila subsp. pneumophila str. Philadelphia 1] |
| ZT1000340 | 1.40E-32 | 137.5 | VFG038724(gi:145298370) | (ASA_1361) SOJ-like and chromosome partitioning protein [Polar flagella (CVF786)] [Aeromonas salmonicida subsp. salmonicida A449] |
| ZT1002493 | 2.40E-32 | 136.7 | VFG014128(gi:152985579) | (PSPA7_1979) imidazole glycerol phosphate synthase subunit HisF [LPS O-antigen (P. aeruginosa) (CVF520)] [Pseudomonas aeruginosa PA7] |
| ZT1002786 | 7.70E-32 | 136.3 | VFG031151(gi:120404430) | (mpa) vesicle-fusing ATPase [Proteasome-associated proteins (CVF656)] [Mycobacterium vanbaalenii PYR-1] |
| ZT1001884 | 7.30E-32 | 136.3 | VFG043040(gi:16765261) | (tar/cheM) methyl accepting chemotaxis protein II [peritrichous flagella (AI139)] [Salmonella enterica subsp. enterica serovar Typhimurium str. LT2] |
| ZT1001944 | 1.10E-31 | 134.8 | VFG044083(gb\|NP_250948) | (ptxR) transcriptional regulator PtxR [pyoverdine (IA001)] [Pseudomonas aeruginosa PAO1] |
| ZT1002495 | 7.00E-32 | 134.8 | VFG045298(gi:52840987) | (hisH) imidazole glycerol phosphate synthase subunit HisH [LPS (VF0171)] [Legionella pneumophila subsp. pneumophila str. Philadelphia 1] |
| ZT1000822 | 1.20E-31 | 134.8 | VFG030700(gi:507420313) | (sugC) sugar ABC transporter ATP-binding protein SugC [Trehalose-recycling ABC transporter (CVF651)] [Mycobacterium abscessus subsp. bolletii 50594] |
| ZT1001925 | 1.50E-31 | 134 | VFG038841(gi:145297258) | (flmH) 3-oxoacyl-ACP reductase [Polar flagella (CVF786)] [Aeromonas salmonicida subsp. salmonicida A449] |
| ZT1002384 | 6.60E-32 | 134 | VFG015701(gi:170722846) | (fliN) flagellar motor switch protein FliN [Flagella (CVF521)] [Pseudomonas putida W619] |
| ZT1003439 | 4.90E-31 | 133.7 | VFG030387(gi:433645508) | (tgs4) acyl-CoA synthetase (AMP-forming)/AMP-acid ligase II [MymA operon (CVF649)] [Mycobacterium smegmatis JS623] |
| ZT1002933 | 2.40E-31 | 133.3 | VFG045705(gi:289165397) | (mip) macrophage infectivity potentiator [Mip (CVF349)] [Legionella longbeachae NSW150] |
| ZT1003673 | 1.40E-31 | 133.3 | VFG040895(gi:21230139) | (xpsI) general secretion pathway protein I [xps (SS213)] [Xanthomonas campestris pv. campestris str. ATCC 33913] |
| ZT1003229 | 5.20E-31 | 132.5 | VFG007911(gi:15828268) | (ddrA) probable antibiotic resistance efflux protein [PDIM (phthiocerol dimycocerosate) and PGL (phenolic glycolipid) biosynthesis and transport (CVF288)] [Mycobacterium leprae TN] |
| ZT1001268 | 8.10E-31 | 132.5 | VFG036960(gi:194099566) | (farB) efflux pump protein FarB [FarAB (CVF758)] [Neisseria gonorrhoeae NCCP11945] |
| ZT1001840 | 9.60E-31 | 132.1 | VFG018697(gi:163868782) | (virB10) VirB10 protein [VirB/VirD4 type IV secretion system & translocated effector Beps (CVF603)] [Bartonella tribocorum CIP 105476] |
| ZT1002142 | 5.60E-31 | 132.1 | VFG026443(gi:507422539) | (mgtC) mg2+ transport protein [Magnesium transport (CVF313)] [Mycobacterium abscessus subsp. bolletii 50594] |
| ZT1000882 | 1.30E-30 | 131.7 | VFG000313(gb\|NP_207965) | (gluP) glucose/galactose transporter [LPS (VF0056)] [Helicobacter pylori 26695] |
| ZT1000010 | 9.80E-31 | 131.3 | VFG019722(gi:194099155) | (exbB) ExbB [Ton system (CVF202)] [Neisseria gonorrhoeae NCCP11945] |
| ZT1000044 | 1.70E-30 | 130.6 | VFG005766(gi:25010702) | (cylG) hypothetical protein [Beta-hemolysin/cytolysin (CVF171)] [Streptococcus agalactiae NEM316] |
| ZT1002369 | 1.80E-30 | 130.2 | VFG023572(gi:332159933) | (YE105_C0309) putative two-component response regulator [TTSS (chromosomally encoded) (CVF046)] [Yersinia enterocolitica subsp. palearctica 105.5R(r)] |
| ZT1000464 | 6.90E-30 | 129.4 | VFG027137(gi:443492994) | (phoR) two-component system response phosphate sensor kinase, PhoR [PhoP/R (CVF331)] [Mycobacterium liflandii 128FXT] |
| ZT1000029 | 5.00E-30 | 129.4 | VFG030293(gi:108801614) | (adhD) alcohol dehydrogenase GroES-like protein [MymA operon (CVF649)] [Mycobacterium sp. MCS] |
| ZT1002970 | 9.60E-30 | 129.4 | VFG015379(gi:152986872) | (PSPA7_0144) probable ATP-binding component of ABC transporter [Hcp secretion island-1 encoded type VI secretion system (H-T6SS) (CVF535)] [Pseudomonas aeruginosa PA7] |
| ZT1002942 | 5.50E-30 | 129 | VFG021792(gi:300858408) | (ciuD) iron ABC transporter ATP-binding protein [ciu iron uptake and siderophore biosynthesis system (CVF515)] [Corynebacterium pseudotuberculosis FRC41] |
| ZT1002959 | 5.50E-30 | 129 | VFG021792(gi:300858408) | (ciuD) iron ABC transporter ATP-binding protein [ciu iron uptake and siderophore biosynthesis system (CVF515)] [Corynebacterium pseudotuberculosis FRC41] |
| ZT1000809 | 4.60E-30 | 129 | VFG035923(gi:387604877) | (aatC) ABC transporter ATP-binding protein [ABC transporter for dispersin (CVF737)] [Escherichia coli O44:H18 042] |
| ZT1003397 | 8.30E-30 | 129 | VFG005552(gi:125718786) | (tig/ropA) Trigger factor, putative [Trigger factor (CVF149)] [Streptococcus sanguinis SK36] |
| ZT1002046 | 7.70E-30 | 128.3 | VFG009635(gi:145222747) | (narI) respiratory nitrate reductase, gamma subunit [Nitrate reductase (CVF318)] [Mycobacterium gilvum PYR-GCK] |
| ZT1003533 | 7.50E-30 | 128.3 | VFG037112(gi:385852271) | (msrA/B(pilB)) peptide methionine sulfoxide reductase msrA/msrB [Methionine sulphoxide reductase (CVF762)] [Neisseria meningitidis H44/76] |
| ZT1001448 | 2.80E-29 | 127.9 | VFG013978(gi:148548974) | (fimV) Peptidoglycan-binding LysM [Type IV pili biosynthesis (CVF518)] [Pseudomonas putida F1] |
| ZT1000799 | 1.60E-29 | 127.5 | VFG011221(gi:115421187) | (BAV0086) Putative glycosyl transferase [LPS (CVF380)] [Bordetella avium 197N] |
| ZT1002842 | 1.80E-29 | 127.1 | VFG036556(gi:385854783) | (fbpC) iron(III) ABC transporter ATP-binding protein [ABC transporter (CVF197)] [Neisseria meningitidis M01-240355] |
| ZT1000857 | 3.60E-29 | 126.7 | VFG015349(gi:71734872) | (hopAJ2) type III effector HopAJ2 [P. syringae TTSS effectors (CVF534)] [Pseudomonas syringae pv. phaseolicola 1448A] |
| ZT1003658 | 4.30E-29 | 126.3 | VFG048370 | (Kvar_0793) fimbrial protein [Type 3 fimbriae (CVF848)] [Klebsiella variicola At-22] |
| ZT1002344 | 1.10E-28 | 125.9 | VFG041502(gi:292487025) | (hrpX) sensor kinase HrpX [T3SS (SS004)] [Erwinia amylovora CFBP1430] |
| ZT1003661 | 6.50E-29 | 124.8 | VFG042535(gb\|AAA92618) | (f17d-A) F17 fimbrial major subunit protein [F17 pili (AI037)] [Escherichia coli str. 111KH86] |
| ZT1003755 | 8.90E-29 | 124.8 | VFG037112(gi:385852271) | (msrA/B(pilB)) peptide methionine sulfoxide reductase msrA/msrB [Methionine sulphoxide reductase (CVF762)] [Neisseria meningitidis H44/76] |
| ZT1002197 | 1.20E-28 | 124.4 | VFG038840(gi:507521851) | (flmH) 3-oxoacyl-ACP reductase [Polar flagella (VF0473)] [Aeromonas hydrophila ML09-119] |
| ZT1003305 | 1.10E-28 | 124.4 | VFG007914(gi:120404101) | (ddrA) daunorubicin resistance ABC transporter ATPase subunit [PDIM (phthiocerol dimycocerosate) and PGL (phenolic glycolipid) biosynthesis and transport (CVF288)] [Mycobacterium vanbaalenii PYR-1] |
| ZT1001039 | 3.10E-28 | 124.4 | VFG016490(gi:42560712) | (tuf) translation elongation factor Tu [EF-Tu (CVF587)] [Mycoplasma mycoides subsp. mycoides SC str. PG1] |
| ZT1002740 | 1.70E-28 | 122.9 | VFG008118(gi:397678515) | (panD) aspartate 1-decarboxylase [Pantothenate synthesis (CVF305)] [Mycobacterium abscessus subsp. bolletii str. GO 06] |
| ZT1003798 | 3.50E-28 | 122.9 | VFG014966(gi:28867367) | (algR) alginate biosynthesis regulatory protein AlgR [Alginate regulation (CVF523)] [Pseudomonas syringae pv. tomato str. DC3000] |
| ZT1002412 | 1.40E-27 | 122.5 | VFG043209(gb\|YP_001006778) | (cheD) methyl-accepting chemotaxis protein CheD [peritrichous flagella (AI145)] [Yersinia enterocolitica subsp. enterocolitica 8081] |
| ZT1003566 | 5.00E-28 | 122.1 | VFG036559(gi:313668880) | (fbpC) iron-uptake permease ATP-binding protein [ABC transporter (CVF197)] [Neisseria lactamica 020-06] |
| ZT1002299 | 1.30E-27 | 121.7 | VFG022385(gi:333989305) | (mmaA4) methoxy mycolic acid synthase [Methyltransferase (CVF287)] [Mycobacterium sp. JDM601] |
| ZT1003006 | 1.10E-27 | 121.3 | VFG002189(gb\|NP_816140) | (cpsB) phosphatidate cytidylyltransferase [Capsule (VF0361)] [Enterococcus faecalis V583] |
| ZT1003011 | 7.90E-28 | 120.9 | VFG011402(gi:23502030) | (fabZ) (3R)-hydroxymyristoyl ACP dehydratase [LPS (CVF383)] [Brucella suis 1330] |
| ZT1002995 | 2.20E-27 | 120.2 | VFG044447(gi:15599846) | (cupE4) pili assembly chaperone [CupE fimbriae (AI449)] [Pseudomonas aeruginosa PAO1] |
| ZT1001598 | 2.40E-27 | 119.8 | VFG045715(gi:289165302) | (ccmA) heme exporter protein CcmA [Cytochrome c muturation (ccm) locus (CVF358)] [Legionella longbeachae NSW150] |
| ZT1002716 | 2.80E-27 | 119.8 | VFG030724(gi:433649467) | (sugC) carbohydrate ABC transporter ATP-binding protein, CUT1 family [Trehalose-recycling ABC transporter (CVF651)] [Mycobacterium smegmatis JS623] |
| ZT1002600 | 1.60E-27 | 119.8 | VFG031459(gi:15827771) | (ndk) nucleoside diphosphate kinase [Nucleoside diphosphate kinase (CVF660)] [Mycobacterium leprae TN] |
| ZT1002352 | 2.10E-27 | 119.4 | VFG038805(gi:145298489) | (flgC) flagellar basal body rod protein FlgC [Polar flagella (CVF786)] [Aeromonas salmonicida subsp. salmonicida A449] |
| ZT1003699 | 4.00E-27 | 119.4 | VFG013573(gi:113460924) | (hitC) iron(III) ABC transporter, ATP-binding protein [Haemophilus iron transport locus (CVF501)] [Haemophilus somnus 129PT] |
| ZT1000654 | 4.40E-27 | 119.4 | VFG000477(gi:16766230) | (rpoS) sigma S (sigma 38) factor of RNA polymerase, major sigmafactor during stationary phase [RpoS (VF0112)] [Salmonella enterica subsp. enterica serovar Typhimurium str. LT2] |
| ZT1002411 | 2.70E-26 | 118.2 | VFG043209(gb\|YP_001006778) | (cheD) methyl-accepting chemotaxis protein CheD [peritrichous flagella (AI145)] [Yersinia enterocolitica subsp. enterocolitica 8081] |
| ZT1003055 | 1.10E-26 | 118.2 | VFG036942(gi:194099567) | (farA) Efflux pump protein, fatty acid resistance [FarAB (CVF758)] [Neisseria gonorrhoeae NCCP11945] |
| ZT1001534 | 2.60E-26 | 118.2 | VFG044394(gi:6959518) | (pdtorfK) putative receptor precursor [Pyridine-2,6-dithiocarboxylic acid (PDTC) (IA030)] [Pseudomonas stutzeri KC] |
| ZT1000650 | 1.30E-26 | 117.5 | VFG036556(gi:385854783) | (fbpC) iron(III) ABC transporter ATP-binding protein [ABC transporter (CVF197)] [Neisseria meningitidis M01-240355] |
| ZT1000280 | 2.70E-26 | 116.3 | VFG042668(gi:206564594) | (cblR) two-component regulatory system response regulator protein [cable pilus (AI081)] [Burkholderia cenocepacia J2315] |
| ZT1003938 | 3.40E-26 | 116.3 | VFG013716(gi:68535383) | (hmuV) putative iron ABC transport system, ATP-binding protein [ABC-type heme transporter (CVF514)] [Corynebacterium jeikeium K411] |
| ZT1000990 | 3.30E-26 | 115.9 | VFG037535(gi:407931896) | (hemO) heme oxygenase [Heme utilization (CVF769)] [Acinetobacter baumannii TYTH-1] |
| ZT1002602 | 1.30E-25 | 115.9 | VFG043691(gi:112292716) | (clbD) putative 3-hydroxyacyl-CoA dehydrogenase [colibactin (TX033)] [Escherichia coli O18:K1:H7 str. IHE3034] |
| ZT1000428 | 8.40E-26 | 115.9 | VFG030161(gi:118617025) | (caeA) exported protease [Carboxylesterase (CVF648)] [Mycobacterium ulcerans Agy99] |
| ZT1003679 | 5.50E-25 | 115.5 | VFG042320(gi:122879039) | (XOO0681) outer membrane protein [XadA (SS166)] [Xanthomonas oryzae pv. oryzae KACC10331] |
| ZT1000710 | 7.40E-26 | 115.2 | VFG005766(gi:25010702) | (cylG) hypothetical protein [Beta-hemolysin/cytolysin (CVF171)] [Streptococcus agalactiae NEM316] |
| ZT1003757 | 8.50E-26 | 114.8 | VFG047459 | (bioD) dethiobiotin synthase [Biotin metabolism (CVF838)] [Francisella tularensis subsp. mediasiatica FSC147] |
| ZT1002461 | 1.30E-25 | 114.8 | VFG009819(gi:145222174) | (phoR) integral membrane sensor signal transduction histidine kinase [PhoP/R (CVF331)] [Mycobacterium gilvum PYR-GCK] |
| ZT1001320 | 2.60E-25 | 114.8 | VFG017683(gi:386003509) | (icl) isocitrate lyase [Isocitrate lyase (CVF302)] [Mycobacterium tuberculosis RGTB423] |
| ZT1002241 | 1.40E-25 | 114.8 | VFG030305(gi:507423706) | (adhD) zinc binding alcohol dehydrogenase [MymA operon (CVF649)] [Mycobacterium abscessus subsp. bolletii 50594] |
| ZT1001946 | 1.10E-25 | 114.4 | VFG045728(gi:289165979) | (letA) Legionella transmission activator LetA [LetA/LetS two component (CVF363)] [Legionella longbeachae NSW150] |
| ZT1002522 | 1.30E-25 | 114 | VFG048966 | (KOX_25215) putative acid phosphatase [Capsule (CVF854)] [Klebsiella oxytoca KCTC 1686] |
| ZT1002366 | 2.00E-25 | 112.8 | VFG014330(gb\|NP_249786) | (fliS) flagellar protein FliS [Deoxyhexose linking sugar, 209 Da capping structure (AI138)] [Pseudomonas aeruginosa PAO1] |
| ZT1003445 | 1.30E-24 | 112.1 | VFG043683(gi:112292708) | (clbL) putative amidase [colibactin (TX033)] [Escherichia coli O18:K1:H7 str. IHE3034] |
| ZT1000265 | 4.90E-25 | 112.1 | VFG009892(gi:118472166) | (devR/dosR) two component transcriptional regulatory protein devr [DevR/S (CVF334)] [Mycobacterium smegmatis str. MC2 155] |
| ZT1000171 | 1.30E-24 | 112.1 | VFG030387(gi:433645508) | (tgs4) acyl-CoA synthetase (AMP-forming)/AMP-acid ligase II [MymA operon (CVF649)] [Mycobacterium smegmatis JS623] |
| ZT1001479 | 6.20E-25 | 111.7 | VFG039487(gi:212212196) | (CbuG_0575) hypothetical protein [T4SS effectors (CVF803)] [Coxiella burnetii CbuG_Q212] |
| ZT1000873 | 1.60E-24 | 111.7 | VFG014378(gi:70729027) | (fleS) sensory box histidine kinase FleS [Flagella (CVF521)] [Pseudomonas fluorescens Pf-5] |
| ZT1003318 | 1.30E-24 | 111.3 | VFG047699 | (OOM_0011) aspartate carbamoyltransferase [Pyrimidine biosynthesis (CVF845)] [Francisella noatunensis subsp. orientalis str. Toba 04] |
| ZT1000052 | 5.30E-24 | 110.5 | VFG016188(gi:28869795) | (fyuA) TonB-dependent siderophore receptor, putative [yersiniabactin (IA005)] [Pseudomonas syringae pv. tomato str. DC3000] |
| ZT1000943 | 4.30E-24 | 110.2 | VFG009851(gi:126437396) | (prrB) integral membrane sensor signal transduction histidine kinase [PrrA/B (CVF332)] [Mycobacterium sp. JLS] |
| ZT1002109 | 4.20E-24 | 110.2 | VFG009877(gi:118619771) | (mprB) two component sensor kinase MprB [MprA/B (CVF333)] [Mycobacterium ulcerans Agy99] |
| ZT1001124 | 5.50E-24 | 109.8 | VFG027156(gi:433650274) | (phoR) signal transduction histidine kinase [PhoP/R (CVF331)] [Mycobacterium smegmatis JS623] |
| ZT1002291 | 9.60E-24 | 109.8 | VFG042740(gi:15599489) | (pprA) two-component sensor PprA [type IV pili (AI098)] [Pseudomonas aeruginosa PAO1] |
| ZT1001597 | 3.60E-24 | 109.4 | VFG010866(gb\|YP_094892) | (ccmB) ABC transporter involved in cytochrome c biogenesis, CcmB subunit [Cytochrome c muturation (ccm) locus (CVF358)] [Legionella pneumophila subsp. pneumophila str. Philadelphia 1] |
| ZT1002893 | 4.80E-24 | 109.4 | VFG041578(gi:269138240) | (ETAE_0884) putative transglycosylase signal peptide protein [T3SS (SS025)] [Edwardsiella tarda EIB202] |
| ZT1003010 | 7.00E-24 | 109 | VFG013376(gi:148826441) | (lpxD) UDP-3-O-[3-hydroxymyristoyl] glucosamine N-acyltransferase [LOS (CVF494)] [Haemophilus influenzae PittEE] |
| ZT1001164 | 7.70E-24 | 108.6 | VFG044084(gi:15597579) | (PA2383) transcriptional regulator [pyoverdine (IA001)] [Pseudomonas aeruginosa PAO1] |
| ZT1001903 | 4.50E-24 | 108.6 | VFG044095(gi:15598606) | (hasL) sigma-70 factor, ECF subfamily [HasA-type hemophore-mediated heme uptake system (IA041)] [Pseudomonas aeruginosa PAO1] |
| ZT1001147 | 2.80E-23 | 108.2 | VFG048540 | (KOX_18265) outer membrane receptor FepA [Salmochelin (CVF850)] [Klebsiella oxytoca KCTC 1686] |
| ZT1002069 | 1.10E-23 | 108.2 | VFG044156(gi:70732684) | (hasS) sigma factor regulatory protein FecR/PupR family [HasA-type hemophore-mediated heme uptake system (IA042)] [Pseudomonas fluorescens Pf-5] |
| ZT1002098 | 1.20E-23 | 107.8 | VFG037505(gi:184157196) | (ACICU_00876) hypothetical protein [Heme utilization (CVF769)] [Acinetobacter baumannii ACICU] |
| ZT1000056 | 1.20E-23 | 107.8 | VFG013730(gi:38233653) | (fagC) Putative iron-siderophore uptake system ATP-binding component [ABC transporter (CVF516)] [Corynebacterium diphtheriae NCTC 13129] |
| ZT1002346 | 1.90E-23 | 107.5 | VFG042740(gi:15599489) | (pprA) two-component sensor PprA [type IV pili (AI098)] [Pseudomonas aeruginosa PAO1] |
| ZT1000310 | 5.50E-23 | 107.1 | VFG043209(gb\|YP_001006778) | (cheD) methyl-accepting chemotaxis protein CheD [peritrichous flagella (AI145)] [Yersinia enterocolitica subsp. enterocolitica 8081] |
| ZT1003753 | 1.50E-23 | 107.1 | VFG037104(gi:194099662) | (msrA/B(pilB)) trifunctional thioredoxin/methionine sulfoxide reductase A/B protein [Methionine sulphoxide reductase (CVF762)] [Neisseria gonorrhoeae NCCP11945] |
| ZT1003589 | 1.90E-23 | 107.1 | VFG014966(gi:28867367) | (algR) alginate biosynthesis regulatory protein AlgR [Alginate regulation (CVF523)] [Pseudomonas syringae pv. tomato str. DC3000] |
| ZT1001057 | 1.70E-23 | 106.7 | VFG042673(gi:206564599) | (cblA) giant cable pilus [cable pilus (AI081)] [Burkholderia cenocepacia J2315] |
| ZT1002114 | 2.60E-23 | 106.7 | VFG005766(gi:25010702) | (cylG) hypothetical protein [Beta-hemolysin/cytolysin (CVF171)] [Streptococcus agalactiae NEM316] |
| ZT1003509 | 5.80E-23 | 106.7 | VFG007283(gi:37677125) | (hap/vvp) Zinc metalloprotease, vibriolysin [Metalloproteases (CVF279)] [Vibrio vulnificus YJ016] |
| ZT1000328 | 1.50E-22 | 106.3 | VFG002045(gi:33592925) | (bvgS) virulence sensor protein [BvgAS (VF0336)] [Bordetella pertussis Tohama I] |
| ZT1000829 | 3.70E-23 | 105.1 | VFG037541(gi:169633822) | (ABSDF2280) hypothetical protein [Heme utilization (CVF769)] [Acinetobacter baumannii SDF] |
| ZT1000158 | 1.10E-22 | 104.4 | VFG045728(gi:289165979) | (letA) Legionella transmission activator LetA [LetA/LetS two component (CVF363)] [Legionella longbeachae NSW150] |
| ZT1003198 | 3.80E-22 | 103.6 | VFG038240(gi:469820883) | (bfmS) sensory histidine kinase in two-component regulatory system with RstA [Two-component system (CVF778)] [Acinetobacter baumannii D1279779] |
| ZT1001059 | 1.60E-22 | 103.6 | VFG042671(gi:206564597) | (cblD) putative minor pilin and initiator [cable pilus (AI081)] [Burkholderia cenocepacia J2315] |
| ZT1001901 | 3.80E-22 | 103.2 | VFG044073(gi:218561857) | (ctuA) putative TonB-denpendent outer membrane receptor [Transferrin and lactoferrin-mediated iron uptake (IA066)] [Campylobacter jejuni subsp. jejuni NCTC 11168] |
| ZT1002532 | 5.40E-22 | 102.8 | VFG002184(gb\|NP_816135) | (cpsG) MurB family protein [Capsule (VF0361)] [Enterococcus faecalis V583] |
| ZT1003929 | 4.50E-22 | 102.8 | VFG036942(gi:194099567) | (farA) Efflux pump protein, fatty acid resistance [FarAB (CVF758)] [Neisseria gonorrhoeae NCCP11945] |
| ZT1000461 | 3.40E-22 | 102.4 | VFG045340(gb\|NP_539653) | (ricA) Rab2 interacting conserved protein A [RicA (VF0414)] [Brucella melitensis bv. 1 str. 16M] |
| ZT1000929 | 3.40E-22 | 102.4 | VFG039427(gi:212218523) | (CbuK_0945) hypothetical protein [T4SS effectors (CVF803)] [Coxiella burnetii CbuK_Q154] |
| ZT1002857 | 8.60E-22 | 102.4 | VFG022949(gi:333989573) | (mprB) two-component sensor kinase MprB [MprA/B (CVF333)] [Mycobacterium sp. JDM601] |
| ZT1002123 | 4.40E-22 | 102.4 | VFG015379(gi:152986872) | (PSPA7_0144) probable ATP-binding component of ABC transporter [Hcp secretion island-1 encoded type VI secretion system (H-T6SS) (CVF535)] [Pseudomonas aeruginosa PA7] |
| ZT1000392 | 7.60E-22 | 102.4 | VFG015866(gi:28871813) | (cfa3) coronafacic acid beta-ketoacyl synthetase component [Phytotoxin coronatine (CVF545)] [Pseudomonas syringae pv. tomato str. DC3000] |
| ZT1001129 | 4.70E-22 | 101.7 | VFG010459(gb\|YP_095932) | (pilE/pilA) type IV pilus assembly protein, major pilin [type IV pili (AI111)] [Legionella pneumophila subsp. pneumophila str. Philadelphia 1] |
| ZT1003751 | 1.60E-21 | 101.7 | VFG031693(gi:315442359) | (senX3) histidine kinase [SenX3 (CVF666)] [Mycobacterium gilvum Spyr1] |
| ZT1003715 | 1.20E-21 | 101.3 | VFG049038 | (KPN2242_15480) hypothetical protein [LPS rfb locus (CVF857)] [Klebsiella pneumoniae KCTC 2242] |
| ZT1003140 | 7.50E-22 | 101.3 | VFG006813(gi:116873279) | (lspA) lipoprotein signal peptidase [Lipoprotein-specific signal peptidase II (CVF249)] [Listeria welshimeri serovar 6b str. SLCC5334] |
| ZT1003761 | 4.50E-21 | 100.5 | VFG007263(gi:15640502) | (irgA) iron-regulated outer membrane virulence protein, TonB receptor family [Enterobactin receptors (CVF277)] [Vibrio cholerae O1 biovar El Tor str. N16961] |
| ZT1002351 | 1.20E-21 | 100.1 | VFG014149(gi:152985328) | (flgB) flagellar basal-body rod protein FlgB [Flagella (CVF521)] [Pseudomonas aeruginosa PA7] |
| ZT1002084 | 1.80E-21 | 99.8 | VFG040870(gi:15595875) | (hxcU) HxcU pseudopilin [hxc (SS204)] [Pseudomonas aeruginosa PAO1] |
| ZT1002695 | 2.90E-21 | 99.8 | VFG043482(gi:70729258) | (oprF) outer membrane protein OprF [Fibronectin-binding protein (AI231)] [Pseudomonas fluorescens Pf-5] |
| ZT1001986 | 6.90E-21 | 99 | VFG022441(gi:183981780) | (mas) multifunctional mycocerosic acid synthase membrane-associated Mas [PDIM (phthiocerol dimycocerosate) and PGL (phenolic glycolipid) biosynthesis and transport (CVF288)] [Mycobacterium marinum M] |
| ZT1000883 | 9.90E-21 | 98.6 | VFG002197(gb\|NP_814691) | (bopD) sugar-binding transcriptional regulator, LacI family [BopD (VF0362)] [Enterococcus faecalis V583] |
| ZT1002024 | 1.70E-20 | 98.2 | VFG037745(gi:407933448) | (adeH) NodT family efflux transporter outer membrane lipoprotein [AdeFGH efflux pump/transport autoinducer (CVF773)] [Acinetobacter baumannii TYTH-1] |
| ZT1000297 | 4.20E-20 | 97.8 | VFG009879(gi:145222478) | (mprB) integral membrane sensor signal transduction histidine kinase [MprA/B (CVF333)] [Mycobacterium gilvum PYR-GCK] |
| ZT1001902 | 1.90E-20 | 97.4 | VFG044087(gi:15597584) | (fpvR) FpvR [pyoverdine (IA001)] [Pseudomonas aeruginosa PAO1] |
| ZT1001881 | 4.50E-20 | 97.4 | VFG043139(gi:28901366) | (scrC) sensory box/GGDEF family protein SrcC [lateral flagella (AI142)] [Vibrio parahaemolyticus RIMD 2210633] |
| ZT1003077 | 3.70E-20 | 97.1 | VFG027203(gi:499077337) | (prrB) two component system sensor histidine kinase prrB [PrrA/B (CVF332)] [Mycobacterium avium subsp. paratuberculosis MAP4] |
| ZT1002227 | 3.80E-20 | 97.1 | VFG044370(gi:33521628) | (qbsN) QbsN [thioquinolobactin (IA009)] [Pseudomonas fluorescens ATCC 17400] |
| ZT1003524 | 5.00E-20 | 96.7 | VFG044351(gi:307130374) | (yhcA) Inner membrane protein of tripartite multidrug resistance system [achromobactin (IA011)] [Dickeya dadantii 3937] |
| ZT1002529 | 6.10E-20 | 95.9 | VFG006083(gi:116628180) | (STER_1441) Glycosyltransferase involved in cell wall biogenesis [Capsule (CVF186)] [Streptococcus thermophilus LMD-9] |
| ZT1000818 | 6.30E-20 | 95.5 | VFG038841(gi:145297258) | (flmH) 3-oxoacyl-ACP reductase [Polar flagella (CVF786)] [Aeromonas salmonicida subsp. salmonicida A449] |
| ZT1003039 | 1.30E-19 | 95.5 | VFG043139(gi:28901366) | (scrC) sensory box/GGDEF family protein SrcC [lateral flagella (AI142)] [Vibrio parahaemolyticus RIMD 2210633] |
| ZT1000662 | 1.00E-19 | 95.1 | VFG002189(gb\|NP_816140) | (cpsB) phosphatidate cytidylyltransferase [Capsule (VF0361)] [Enterococcus faecalis V583] |
| ZT1000287 | 7.70E-20 | 95.1 | VFG038840(gi:507521851) | (flmH) 3-oxoacyl-ACP reductase [Polar flagella (VF0473)] [Aeromonas hydrophila ML09-119] |
| ZT1002407 | 3.90E-19 | 94.4 | VFG043209(gb\|YP_001006778) | (cheD) methyl-accepting chemotaxis protein CheD [peritrichous flagella (AI145)] [Yersinia enterocolitica subsp. enterocolitica 8081] |
| ZT1002349 | 1.10E-19 | 94.4 | VFG015720(gi:167034950) | (flgA) flagella basal body P-ring formation protein FlgA [Flagella (CVF521)] [Pseudomonas putida GB-1] |
| ZT1001961 | 1.60E-19 | 94.4 | VFG042747(gi:15599496) | (tadC) TadC [type IV pili (AI098)] [Pseudomonas aeruginosa PAO1] |
| ZT1002313 | 2.40E-19 | 94 | VFG044238(gi:123440715) | (hemT) hemin-binding periplasmic protein [direct heme uptake system (IA059)] [Yersinia enterocolitica subsp. enterocolitica 8081] |
| ZT1000406 | 6.30E-19 | 93.6 | VFG043209(gb\|YP_001006778) | (cheD) methyl-accepting chemotaxis protein CheD [peritrichous flagella (AI145)] [Yersinia enterocolitica subsp. enterocolitica 8081] |
| ZT1002576 | 2.30E-19 | 93.6 | VFG019276(gi:163844212) | (dhbA) hypothetical protein [Brucebactin (CVF386)] [Brucella suis ATCC 23445] |
| ZT1002950 | 5.50E-19 | 93.2 | VFG038238(gi:523529861) | (bfmS) Signal transduction histidine kinase [Two-component system (CVF778)] [Acinetobacter baumannii BJAB0715] |
| ZT1002967 | 5.50E-19 | 93.2 | VFG038238(gi:523529861) | (bfmS) Signal transduction histidine kinase [Two-component system (CVF778)] [Acinetobacter baumannii BJAB0715] |
| ZT1003430 | 4.20E-19 | 93.2 | VFG043482(gi:70729258) | (oprF) outer membrane protein OprF [Fibronectin-binding protein (AI231)] [Pseudomonas fluorescens Pf-5] |
| ZT1003097 | 1.10E-18 | 92.8 | VFG043209(gb\|YP_001006778) | (cheD) methyl-accepting chemotaxis protein CheD [peritrichous flagella (AI145)] [Yersinia enterocolitica subsp. enterocolitica 8081] |
| ZT1002405 | 1.80E-19 | 92.8 | VFG025824(gi:107024388) | (cheY) response regulator receiver protein [Flagella (CVF643)] [Burkholderia cenocepacia AU 1054] |
| ZT1002623 | 1.20E-18 | 92.4 | VFG013719(gi:62389290) | (hmuV) cobalamin/Fe3+-siderophores transport system, ATPase component [ABC-type heme transporter (CVF514)] [Corynebacterium glutamicum ATCC 13032 (DSM 20300)] |
| ZT1003248 | 5.80E-19 | 92.4 | VFG015519(gi:152986215) | (phzF1) probable phenazine biosynthesis protein [Phenazines biosynthesis (CVF536)] [Pseudomonas aeruginosa PA7] |
| ZT1002095 | 5.90E-19 | 92.4 | VFG044156(gi:70732684) | (hasS) sigma factor regulatory protein FecR/PupR family [HasA-type hemophore-mediated heme uptake system (IA042)] [Pseudomonas fluorescens Pf-5] |
| ZT1002080 | 4.20E-18 | 91.7 | VFG034541(gb\|YP_006203830) | (etpA) Two-partner secreted adhesin EtpA [EtpA (VF0437)] [Escherichia coli O78:H11:K80 str. H10407] |
| ZT1000473 | 8.80E-19 | 91.7 | VFG019276(gi:163844212) | (dhbA) hypothetical protein [Brucebactin (CVF386)] [Brucella suis ATCC 23445] |
| ZT1003856 | 8.00E-19 | 91.7 | VFG043388(gi:15645899) | (fliZ) hypothetical protein [Pse5Ac7Ac (AI150)] [Helicobacter pylori 26695] |
| ZT1000291 | 7.10E-19 | 91.7 | VFG010911(gi:54298635) | (letA) Legionella transmission activator LetA [LetA/LetS two component (CVF363)] [Legionella pneumophila str. Paris] |
| ZT1001828 | 1.90E-18 | 91.7 | VFG042740(gi:15599489) | (pprA) two-component sensor PprA [type IV pili (AI098)] [Pseudomonas aeruginosa PAO1] |
| ZT1003215 | 1.20E-18 | 91.7 | VFG043139(gi:28901366) | (scrC) sensory box/GGDEF family protein SrcC [lateral flagella (AI142)] [Vibrio parahaemolyticus RIMD 2210633] |
| ZT1003361 | 1.30E-18 | 91.3 | VFG007856(gi:126435444) | (ppsC) beta-ketoacyl synthase [PDIM (phthiocerol dimycocerosate) and PGL (phenolic glycolipid) biosynthesis and transport (CVF288)] [Mycobacterium sp. JLS] |
| ZT1000488 | 2.60E-18 | 91.3 | VFG044370(gi:33521628) | (qbsN) QbsN [thioquinolobactin (IA009)] [Pseudomonas fluorescens ATCC 17400] |
| ZT1003061 | 2.80E-18 | 90.9 | VFG022933(gi:333989462) | (prrB) two-component sensor histidine kinase [PrrA/B (CVF332)] [Mycobacterium sp. JDM601] |
| ZT1002329 | 7.20E-19 | 90.5 | VFG016799(gi:49475465) | (vapA3) Virulence-associated protein A [Virulence-associated proteins (CVF607)] [Bartonella henselae str. Houston-1] |
| ZT1000804 | 3.40E-18 | 90.5 | VFG014378(gi:70729027) | (fleS) sensory box histidine kinase FleS [Flagella (CVF521)] [Pseudomonas fluorescens Pf-5] |
| ZT1000331 | 1.70E-18 | 90.5 | VFG021188(gi:194448069) | (fimZ) transcriptional regulator FimZ [Fim (CVF003)] [Salmonella enterica subsp. enterica serovar Heidelberg str. SL476] |
| ZT1002516 | 1.90E-18 | 90.1 | VFG007550(gi:15642062) | (cheB) chemotaxis-specific methylesterase [Flagella (VF0519)] [Vibrio cholerae O1 biovar El Tor str. N16961] |
| ZT1002774 | 8.60E-19 | 89.7 | VFG010907(gi:54296805) | (csrA) global regulator CsrA [Carbon storage regulator A (CVF362)] [Legionella pneumophila str. Paris] |
| ZT1003359 | 1.30E-18 | 89.4 | VFG011430(gb\|NP_540392) | (acpXL) acyl carrier protein [LPS (CVF383)] [Brucella melitensis bv. 1 str. 16M] |
| ZT1001297 | 1.20E-17 | 89.4 | VFG025888(gi:83720865) | (tsr) methyl-accepting chemotaxis protein [Flagella (CVF643)] [Burkholderia thailandensis E264] |
| ZT1002097 | 5.20E-18 | 89 | VFG037505(gi:184157196) | (ACICU_00876) hypothetical protein [Heme utilization (CVF769)] [Acinetobacter baumannii ACICU] |
| ZT1002321 | 1.70E-17 | 89 | VFG018161(gi:49482524) | (essC) hypothetical protein [Type VII secretion system (CVF624)] [Staphylococcus aureus subsp. aureus MRSA252] |
| ZT1003432 | 1.00E-17 | 88.6 | VFG026090(gi:170732104) | (wcbR) Beta-ketoacyl synthase [Capsule I (CVF645)] [Burkholderia cenocepacia MC0-3] |
| ZT1001102 | 1.10E-17 | 88.6 | VFG049129 | (KPHS_11890) acridine efflux pump [AcrAB (CVF859)] [Klebsiella pneumoniae subsp. pneumoniae HS11286] |
| ZT1003392 | 2.40E-17 | 88.2 | VFG043537(gi:218562247) | (Cj0596) major antigenic peptide PEB-cell binding factor [Peb4/CBF2 (AI315)] [Campylobacter jejuni subsp. jejuni NCTC 11168] |
| ZT1002191 | 2.00E-17 | 87.8 | VFG042320(gi:122879039) | (XOO0681) outer membrane protein [XadA (SS166)] [Xanthomonas oryzae pv. oryzae KACC10331] |
| ZT1000986 | 1.10E-17 | 87.4 | VFG044157(gi:70732685) | (hasL) ECF subfamily RNA polymerase sigma factor [HasA-type hemophore-mediated heme uptake system (IA042)] [Pseudomonas fluorescens Pf-5] |
| ZT1003646 | 4.60E-17 | 87 | VFG037388(gi:169795230) | (bauE) ferric acinetobactin transport system ATP-binding protein [Acinetobactin (CVF768)] [Acinetobacter baumannii AYE] |
| ZT1000945 | 2.40E-17 | 86.7 | VFG041501(gi:292487026) | (hrpY) response regulator hrpY [T3SS (SS004)] [Erwinia amylovora CFBP1430] |
| ZT1002868 | 1.50E-17 | 86.7 | VFG042948(gi:28199487) | (pilE) type IV pilin [type IV pili (AI118)] [Xylella fastidiosa Temecula1] |
| ZT1003171 | 3.80E-17 | 86.3 | VFG018030(gi:157414377) | (flhG) putative ATP-binding protein [Flagella (CVF397)] [Campylobacter jejuni subsp. jejuni 81116] |
| ZT1003208 | 7.30E-17 | 86.3 | VFG019157(gi:153940999) | (CLI_2965) transporter, HlyC/CorC family [Hemolysin (CVF417)] [Clostridium botulinum F str. Langeland] |
| ZT1002919 | 1.10E-16 | 85.5 | VFG043139(gi:28901366) | (scrC) sensory box/GGDEF family protein SrcC [lateral flagella (AI142)] [Vibrio parahaemolyticus RIMD 2210633] |
| ZT1003270 | 3.20E-16 | 84.7 | VFG044319(gi:206560062) | (orbA) ornibactin receptor [ornibactin (IA036)] [Burkholderia cenocepacia J2315] |
| ZT1001675 | 1.20E-16 | 84.7 | VFG008680(gi:433650447) | (ddrA) daunorubicin resistance ABC transporter ATP-binding subunit [PDIM (phthiocerol dimycocerosate) and PGL (phenolic glycolipid) biosynthesis and transport (CVF288)] [Mycobacterium smegmatis JS623] |
| ZT1001079 | 5.50E-17 | 84.3 | VFG018756(gi:163869119) | (vapA5) virulence-associated protein [Virulence-associated proteins (CVF607)] [Bartonella tribocorum CIP 105476] |
| ZT1001972 | 2.00E-16 | 84 | VFG013216(gi:68249712) | (ompP5) Outer membrane protein P5 [P5 protein (CVF492)] [Haemophilus influenzae 86-028NP] |
| ZT1002517 | 1.90E-16 | 84 | VFG043254(gi:16802725) | (cheR) hypothetical protein [<beta>-GlcNAc (AI147)] [Listeria monocytogenes EGD-e] |
| ZT1002181 | 2.20E-16 | 84 | VFG044084(gi:15597579) | (PA2383) transcriptional regulator [pyoverdine (IA001)] [Pseudomonas aeruginosa PAO1] |
| ZT1000647 | 5.40E-16 | 83.6 | VFG020864(gi:194450969) | (ssaN) type III secretion system ATPase [TTSS (SPI-2 encode) (CVF032)] [Salmonella enterica subsp. enterica serovar Heidelberg str. SL476] |
| ZT1000634 | 5.00E-16 | 83.2 | VFG036983(gi:385340565) | (mtrC) membrane fusion protein MtrC [MtrCDE (CVF759)] [Neisseria meningitidis G2136] |
| ZT1001820 | 5.10E-16 | 82.8 | VFG042369(gi:33578091) | (emaA) EmaA [extracellular matrix protein adhesin A (EmaA) (SS171)] [Actinobacillus actinomycetemcomitans VT1169] |
| ZT1001163 | 9.80E-16 | 82.8 | VFG044091(gi:15598602) | (hasD) transport protein HasD [HasA-type hemophore-mediated heme uptake system (IA041)] [Pseudomonas aeruginosa PAO1] |
| ZT1001821 | 5.40E-16 | 82.4 | VFG041157(gi:206558758) | (bscA) subfamily M15C metalopeptidase [T6SS (SS193)] [Burkholderia cenocepacia J2315] |
| ZT1000570 | 1.10E-15 | 82.4 | VFG043139(gi:28901366) | (scrC) sensory box/GGDEF family protein SrcC [lateral flagella (AI142)] [Vibrio parahaemolyticus RIMD 2210633] |
| ZT1001159 | 1.50E-15 | 82 | VFG030145(gi:392387860) | (kefB) hypothetical protein [Potassium/proton antiporter (CVF647)] [Mycobacterium tuberculosis UT205] |
| ZT1001481 | 2.40E-15 | 82 | VFG016491(gi:26553484) | (tuf) elongation factor Tu [EF-Tu (CVF587)] [Mycoplasma penetrans HF-2] |
| ZT1000958 | 1.10E-15 | 81.6 | VFG011765(gi:118475340) | (CFF8240_1400) acyltransferase [LOS (CVF396)] [Campylobacter fetus subsp. fetus 82-40] |
| ZT1002375 | 4.20E-16 | 81.6 | VFG019830(gi:229591854) | (fliE) flagellar biosynthesis; basal-body component [Flagella (CVF521)] [Pseudomonas fluorescens SBW25] |
| ZT1001966 | 1.20E-15 | 81.6 | VFG042752(gi:15599501) | (rcpC) RcpC [type IV pili (AI098)] [Pseudomonas aeruginosa PAO1] |
| ZT1002338 | 2.40E-15 | 80.9 | VFG015638(gi:167036028) | (pilJ) methyl-accepting chemotaxis sensory transducer [Type IV pili twitching motility related proteins (CVF519)] [Pseudomonas putida GB-1] |
| ZT1002206 | 2.40E-15 | 80.5 | VFG044083(gb\|NP_250948) | (ptxR) transcriptional regulator PtxR [pyoverdine (IA001)] [Pseudomonas aeruginosa PAO1] |
| ZT1003590 | 2.70E-15 | 80.5 | VFG014983(gi:146280917) | (algZ) alginate biosynthesis protein AlgZ/FimS [Alginate regulation (CVF523)] [Pseudomonas stutzeri A1501] |
| ZT1002850 | 1.70E-15 | 80.1 | VFG048933 | (KOX_25200) protein tyrosine phosphatase [Capsule (CVF854)] [Klebsiella oxytoca KCTC 1686] |
| ZT1000234 | 4.90E-15 | 80.1 | VFG022917(gi:333992253) | (phoR) two-component system response phosphate sensor kinase PhoR [PhoP/R (CVF331)] [Mycobacterium sp. JDM601] |
| ZT1000994 | 2.40E-15 | 80.1 | VFG044407(gi:34787216) | (hasB) TonB-like protein [HasA-type hemophore-mediated heme uptake system (IA043)] [Serratia marcescens str. SM365] |
