# Supplementary material for: The complete genome sequence of a bile-isolated Stenotrophomonas maltophilia ZT1
Source: Gut Pathog. 2021 Oct 28;13:64. doi: 10.1186/s13099-021-00456-y (PMC8555292; doi:10.1186/s13099-021-00456-y)
Supplement: Supplementary file 2 — Additional file 2. Table S2. The efflux pump associated virulence factor in S. maltophilia ZT1 predicted by VFDB. [file 13099_2021_456_MOESM2_ESM.docx]

Table S2 The efflux pump associated virulence factor in *S. maltophilia* ZT1 predicted by VFDB

| Gene ID | Gene name | Fuction/ putative fuction | Score | E value |
| --- | --- | --- | --- | --- |
| ZT1002690 | adeG | RND cation/multidrug efflux pump | 1134 | 0.00E+00 |
| ZT1002458 | mtrD | drug efflux protein | 916.4 | 1.90E-266 |
| ZT1001103 | adeG | RND cation/multidrug efflux pump | 493.8 | 3.10E-139 |
| ZT1000633 | adeG | RND cation/multidrug efflux pump | 347.1 | 4.80E-95 |
| ZT1000838 | acrAB | acridine efflux pump | 284.3 | 1.40E-76 |
| ZT1002619 | adeG | RND cation/multidrug efflux pump | 260.4 | 6.50E-69 |
| ZT1000467 | mtrE | multidrug efflux pump channel protein | 253.8 | 2.40E-67 |
| ZT1002979 | farA | Efflux pump protein, fatty acid resistance | 248.8 | 6.50E-66 |
| ZT1001691 | mtrC | antibiotic resistance efflux pump component | 224.9 | 1.10E-58 |
| ZT1002691 | adeF | multidrug ABC transporter | 211.5 | 1.20E-54 |
| ZT1002688 | adeH | Outer membrane protein | 183.3 | 4.10E-46 |
| ZT1002459 | mtrC | (antibiotic resistance efflux pump component | 183.3 | 3.20E-46 |
| ZT1001268 | farB | efflux pump protein | 132.5 | 8.10E-31 |
| ZT1003055 | farA | Efflux pump protein, fatty acid resistance | 118.2 | 1.10E-26 |
| ZT1003929 | farA | Efflux pump protein, fatty acid resistance | 102.8 | 4.50E-22 |
| ZT1002024 | adeH | NodT family efflux transporter outer membrane lipoprotein | 98.2 | 1.70E-20 |
| ZT1001102 | acrAB | acridine efflux pump | 88.6 | 1.10E-17 |
