# Supplementary material for: The complete genome sequence of a bile-isolated Stenotrophomonas maltophilia ZT1
Source: Gut Pathog. 2021 Oct 28;13:64. doi: 10.1186/s13099-021-00456-y (PMC8555292; doi:10.1186/s13099-021-00456-y)
Supplement: Supplementary file 3 — Additional file 3. Table S3. The secretion system associated virulence factor in S. maltophilia ZT1 predicted by VFDB. [file 13099_2021_456_MOESM3_ESM.docx]

Table S3 The secretion system associated virulence factor in *S. maltophilia* ZT1 predicted by VFDB

| Gene ID | Gene name | Fuction/ putative fuction | Score | E value |
| --- | --- | --- | --- | --- |
| ZT1003667 | xpsD | general secretion pathway protein D | 903.7 | 9.10E-263 |
| ZT1003677 | xpsE | general secretion pathway protein E | 884.4 | 4.50E-257 |
| ZT1002076 | hxcQ | type II secretion system protein | 706.8 | 1.80E-203 |
| ZT1002077 | hxcR | type II secretion system protein | 654.8 | 4.80E-188 |
| ZT1001845 | virB4 | type IV secretion/conjugal transfer ATPase | 589.3 | 4.20E-168 |
| ZT1003676 | xpsF | general secretion pathway protein F | 523.9 | 1.10E-148 |
| ZT1002078 | hxcS | type II secretion system protein | 483.8 | 1.20E-136 |
| ZT1003595 | secA2 | protein translocase subunit secA | 431.4 | 1.60E-120 |
| ZT1003670 | xpsL | general secretion pathway protein L | 385.6 | 4.30E-107 |
| ZT1003671 | xpsK | general secretion pathway protein K | 312 | 4.60E-85 |
| ZT1001836 | rvhD4 | type IV secretion system protein VirB8 | 287.7 | 1.80E-77 |
| ZT1001841 | virB11 | P-type DNA transfer ATPase VirB11 | 278.5 | 6.40E-75 |
| ZT1001173 | CBU1566 | Coxiella Dot/Icm type IVB secretion system translocated effector | 255.4 | 4.30E-68 |
| ZT1003669 | xpsM | general secretion pathway protein M | 238.4 | 5.30E-63 |
| ZT1001789 | hlyB | hemolysin secretion protein HlyB | 237.7 | 2.40E-62 |
| ZT1003675 | xpsG | general secretion pathway protein G | 208 | 4.80E-54 |
| ZT1003672 | xpsJ | general secretion pathway protein J | 198.4 | 5.40E-51 |
| ZT1000203 | coxH3 | Coxiella Dot/Icm type IVB secretion system translocated effector | 171.4 | 7.50E-43 |
| ZT1000796 | cdsN | type III secretion system ATPase | 170.2 | 3.50E-42 |
| ZT1003674 | xpsH | general secretion pathway protein H | 168.3 | 4.70E-42 |
| ZT1000794 | hrcN | type III secretion system ATPase | 159.1 | 8.90E-39 |
| ZT1003890 | CBU1594 | Coxiella Dot/Icm type IVB secretion system translocated effector | 158.3 | 4.30E-39 |
| ZT1003668 | xpsN | general secretion pathway protein N | 155.2 | 6.50E-38 |
| ZT1000886 | lpg1661 | Dot/Icm type IV secretion system effector | 153.3 | 3.40E-37 |
| ZT1001842 | virB1 | type IV secretion system protein VirB1 | 148.7 | 7.60E-36 |
| ZT1003386 | pppA | putative phosphatase [Hcp secretion island-1 encoded type VI secretion system] | 146.7 | 2.10E-35 |
| ZT1003442 | lirB | Dot/Icm type IV secretion system effector | 137.5 | 8.80E-33 |
| ZT1003673 | xpsI | general secretion pathway protein I | 133.3 | 1.40E-31 |
| ZT1001840 | virB10 | VirB10 protein [type IV secretion system & translocated effector Beps] | 132.1 | 9.60E-31 |
| ZT1002970 | PSPA7_0144 | probable ATP-binding component of ABC transporter | 129.4 | 9.60E-30 |
| ZT1002123 | PSPA7_0144 | probable ATP-binding component of ABC transporter | 102.4 | 4.40E-22 |
